# *Daf-16* mediated repression of cytosolic ribosomal protein genes facilitates a hypoxia sensitive to hypoxia resistant transformation in long-lived germline mutants

**DOI:** 10.1371/journal.pgen.1009672

**Published:** 2022-05-27

**Authors:** Cassidy Hemphill, Evye Pylarinou-Sinclair, Omar Itani, Barbara Scott, C. Michael Crowder, Marc Ryan Van Gilst

**Affiliations:** 1 Department of Anesthesiology and Pain Medicine, University of Washington, Seattle, Washington, United States of America; 2 Mitochondria and Metabolism Center, University of Washington, Seattle, Washington, United States of America; 3 Department of Genome Sciences, University of Washington, Seattle, Washington, United States of America; University of California San Francisco, UNITED STATES

## Abstract

In *C*. *elegans*, germline ablation leads to long life span and stress resistance. It has been reported that mutations that block oogenesis or an upstream step in germline development confer strong resistance to hypoxia. We demonstrate here that the hypoxia resistance of sterile mutants is dependent on developmental stage and age. In just a 12-hour period, sterile animals transform from hypoxia sensitive L4 larvae into hypoxia resistant adults. Since this transformation occurs in animals with no germline, the physiological programs that determine hypoxia sensitivity in germline mutants occur independently of germline signals and instead rely on signals from somatic tissues. Furthermore, we found two distinct mechanisms of hypoxia resistance in germline deficient animals. First, a DAF-16/FoxO *independent* mechanism that occurs in all hypoxia resistant sterile adults and, second, a DAF-16/FoxO *dependent* mechanism that confers an added layer of resistance, or “super-resistance”, to animals with no germline as they age past day 1 of adulthood. RNAseq data showed that genes involved in both cytosolic and mitochondrial protein translation are repressed in sterile adults and further repressed only in germline deficient mutants as they age. Importantly, mutation of *daf-16* specifically blocked the repression of cytosolic ribosomal protein genes, but not mitochondrial ribosomal protein genes, implicating DAF-16/FoxO mediated repression of cytosolic ribosomal protein genes as a mechanism of hypoxia super-resistance. Consistent with this hypothesis, the hypoxia super-resistance of aging germline deficient adults was also suppressed by dual mutation of *ncl-1* and *larp-1*, two regulators of protein translation and ribosomal protein abundance. These studies provide novel insight into a profound physiological transformation that takes place in germline mutants during development, showing that some of the unique physiological properties of these long-lived animals are derived from developmentally dependent DAF-16/FoxO mediated repression of genes involved in cytosolic protein translation.

## Author summary

In addition to being extremely long lived, germline deficient animals have other extraordinary properties, such as robust resistance to oxygen deprivation. Here we provide new insight into the mechanisms of hypoxia resistance in germline deficient animals. We demonstrate that, in just a 12-hour period, germline mutants transform from hypoxia sensitive larvae into highly hypoxia resistant adults. Therefore, hypoxia resistance is not a universal property of germline ablated animals, but is instead “switched on” only in adult animals. We have found two distinct mechanisms of hypoxia resistance in germline deficient animals and both mechanisms are mediated by signals from somatic tissues and can operate independently of the germline. Using RNA sequencing, we show that *daf-16* is required for the developmental repression of genes involved in cytosolic protein translation and this phenomenon is one of the mechanisms of hypoxia resistance in germline mutants. Consistent with this hypothesis, dual mutation of *ncl-1* and *larp-1*, two regulators of protein translation and cytosolic ribosomal protein levels, also suppressed the hypoxia resistance of germline ablated animals. We conclude that the L4/adult developmental switch presents an excellent opportunity for investigating the mechanisms of longevity and hypoxia resistance in germline deficient animals.

## Introduction

Oxygen is critical for the function of nearly all living organisms. Accordingly, depriving an organism of oxygen can damage or kill cells and tissues and eventually lead to organismal death. In humans, cardiovascular events such as stroke and heart attack impede blood from reaching critical tissues resulting in ischemia and hypoxic injury. It is for this reason that hypoxic injury is intensively studied in a wide range of experimental models, including vertebrate animals, cell culture systems, as well as genetic model organisms such as *C*. *elegans* and *Drosophila* [1–4].

The unique advantage of *C*. *elegans* as an experimental model is its versatile genetic screening capabilities [5]. Well-conceived random mutagenesis or RNAi screens can identify new genes involved in just about any physiological process. Like other animals, depriving *C*. *elegans* of oxygen leads to tissue damage and organismal death, thus nematodes can be an excellent model for studying hypoxic injury [6]. RNAi and mutagenic screens in *C*. *elegans* have identified over 200 genes that, when inhibited, enable animals to better survive oxygen deprivation [1,6,7]. The mechanisms by which most of these genes influence hypoxic injury have yet to be determined.

Perhaps the most well-studied pathways influencing hypoxic injury in *C*. *elegans* are protein translation and insulin signaling [6–8]. The role of protein translation appears to be the most critical, as mutagenesis screens designed to identify genes involved in hypoxic injury isolated multiple genes involved in protein synthesis, far more than any other gene class [7]. Reduced function mutations have been isolated in multiple tRNA synthetase genes, as well as other regulators of protein synthesis such as the RNA helicase gene *ddx-52*, a tRNA nuclear exporter gene *xpo-3*, a tRNA ligase gene *rtcb-1*, and an oligopeptide transporter gene *pept-1*. It has been confirmed that a hypoxia resistant loss of function mutation in the threonine tRNA synthetase *tars-1* leads to a reduction in the rate of protein synthesis [7]. A suppressor screen has isolated genes that can restore translation rates in these reduced translation mutants and has thereby expanded the understanding of regulatory networks that control protein translation and hypoxic injury [7]. Specifically, dual mutations in *larp-1* and *ncl-1*, regulators of protein translation, are able to suppress the hypoxia resistance of several protein translation mutants. Proteomic studies have shown that mutation of *ncl-1* and *larp-1* leads to an increase in ribosomal proteins which enables translation to be restored to wild type levels in tRNA synthetase mutants [7]. In summary, the study of protein translation and its link to hypoxia resistance in *C*. *elegans* has yielded novel insight into both hypoxic injury and protein translation.

Insulin signaling also has a significant impact on hypoxic injury. Specifically, loss of function mutations in the *daf-2* insulin receptor gene lead to strong hypoxia resistance [8]. This occurs through the standard insulin signaling pathway as a loss of function mutation in the FoxO gene homolog *daf-16* is able to suppress the hypoxia resistance of *daf-2* mutants. Mutation of *daf-16* does not suppress the hypoxia resistance of translation mutants, however, arguing that these two pathways influence hypoxia resistance through different mechanisms. Loss of function mutations in *daf-2* also lead to a prolonged lifespan, suggesting there may be a connection between the mechanisms of longevity and hypoxia resistance [9]. However, mutations in protein translation genes that lead to hypoxia resistance have little impact on longevity, implying that hypoxia resistance doesn’t necessarily bring about increases in lifespan [6].

Another factor that has a significant impact on hypoxic injury in *C*. *elegans* is reproduction [10]. Specifically, any sterile mutation that prevents oogenesis or an upstream step in germline development confers strong resistance to hypoxia [10]. For *glp-1(ts)* animals, which do not contain a germline when grown at 25°C, RNAi of AMPK subunits is able to partially suppress hypoxia resistance, potentially linking carbohydrate metabolism to the mechanism of sterile mutant hypoxia resistance [11]. Like *daf-2* mutants, germline ablated animals are long-lived raising the possibility that longevity and hypoxia resistance are related. However, mutation of *daf-16* suppresses the longevity of germline ablated animals whereas it has been reported that mutation of *daf-16* does not suppress the hypoxia resistance of *glp-1(ts)* mutants [10]. Furthermore, other sterile mutants that have not been reported to be long-lived are resistant to hypoxia in a *daf-16* independent fashion, suggesting that longevity and hypoxia resistance are occurring through distinct mechanisms in sterile mutants. The link between sterility and hypoxia resistance is also an important one given that many of the 200+ hypoxia associated genes also partially reduce reproductive output when knocked down [1]. Therefore, knockdown of these genes may yield hypoxia resistance simply because of their impact on reproduction. It is not yet known, however, if partial sterility leads to partial hypoxia resistance, so it is unclear if these genes influence hypoxia by reducing reproductive output.

There is another connection between insulin signaling, germline development, and protein translation. Several long-lived mutants, including *daf-2(lf)* and *glp-1(ts)* mutants, possess smaller nucleoli [12]. In fact, smaller nucleoli, which play a role in the regulation and maturation of ribosomal RNAs, are a hallmark of longevity in multiple species. Consistent with the role of nucleoli in ribosome assembly and protein translation, fewer ribosomal proteins are present in both *daf-2* and *glp-1(ts)* mutants when compared to WT worms [12]. This observation suggests that it is likely that protein translation is reduced in both of these mutants, as well as other long-lived backgrounds. Even though reduced protein translation alone is not sufficient to prolong lifespan, numerous studies have shown that, in some cases, reduced protein translation is involved in lengthened lifespan [13–15]. Mutation of *daf-16* is able to suppress both nucleolar size and longevity in both *daf-2* and *glp-1(ts)* mutants. Smaller nucleoli in both mutants were also suppressed by mutation of *ncl-1*, which influences nucleolar physiology by repressing production of *fib-1*, a regulator of ribosomal RNA maturation [12]. Taken together, there are still many open questions regarding the relationships of reproduction and protein translation to hypoxic injury and longevity. Accordingly, we set out to further establish the mechanisms of hypoxia resistance in sterile mutants.

## Results

### Germline mutants are resistant to hypoxic injury

A previous study has shown that sterile mutations that block oogenesis or an upstream step in germline development render animals resistant to hypoxic injury at 20°C [10]. We set out to identify the mechanisms by which sterility leads to hypoxia resistance in *C*. *elegans*. We selected several different types of sterile strains to determine if mutations that prevent oogenesis also lead to hypoxia resistance under our conditions (because hypoxic injury is more robust and consistent at higher temperatures, we expose our worms to hypoxia at 26.5°C as opposed to 20°C as was previously reported for sterile mutants). The first type of sterile mutations tested included two temperature sensitive mutations, *glp-1(e2141)* and *glp-4(bn2)*. Worms containing either of these two mutations do not develop a germline when grown at or above 25°C. The second type of mutation was a temperature sensitive allele of the *fem-3* gene, *fem-3(q20)*, which leads to a masculinized germline that makes sperm but not oocytes when grown at or above 25°C. The third type of mutation was a different temperature sensitive allele of the *fem-3* gene, *fem-3(e2006)*, which leads to a feminized germline that makes oocytes but not sperm at or above 25°C. The fourth type of mutant was a *gld-1(lf)* mutant that blocks oogenesis by inhibiting stem cell differentiation leading to a tumorous germline full of undifferentiated mitotic cells. Strains containing all of these sterile mutations were tested for hypoxia sensitivity at 26.5°C and compared to wild type (WT) hermaphrodites, which produce both sperm and oocytes under our experimental conditions.

**Fig 1:**
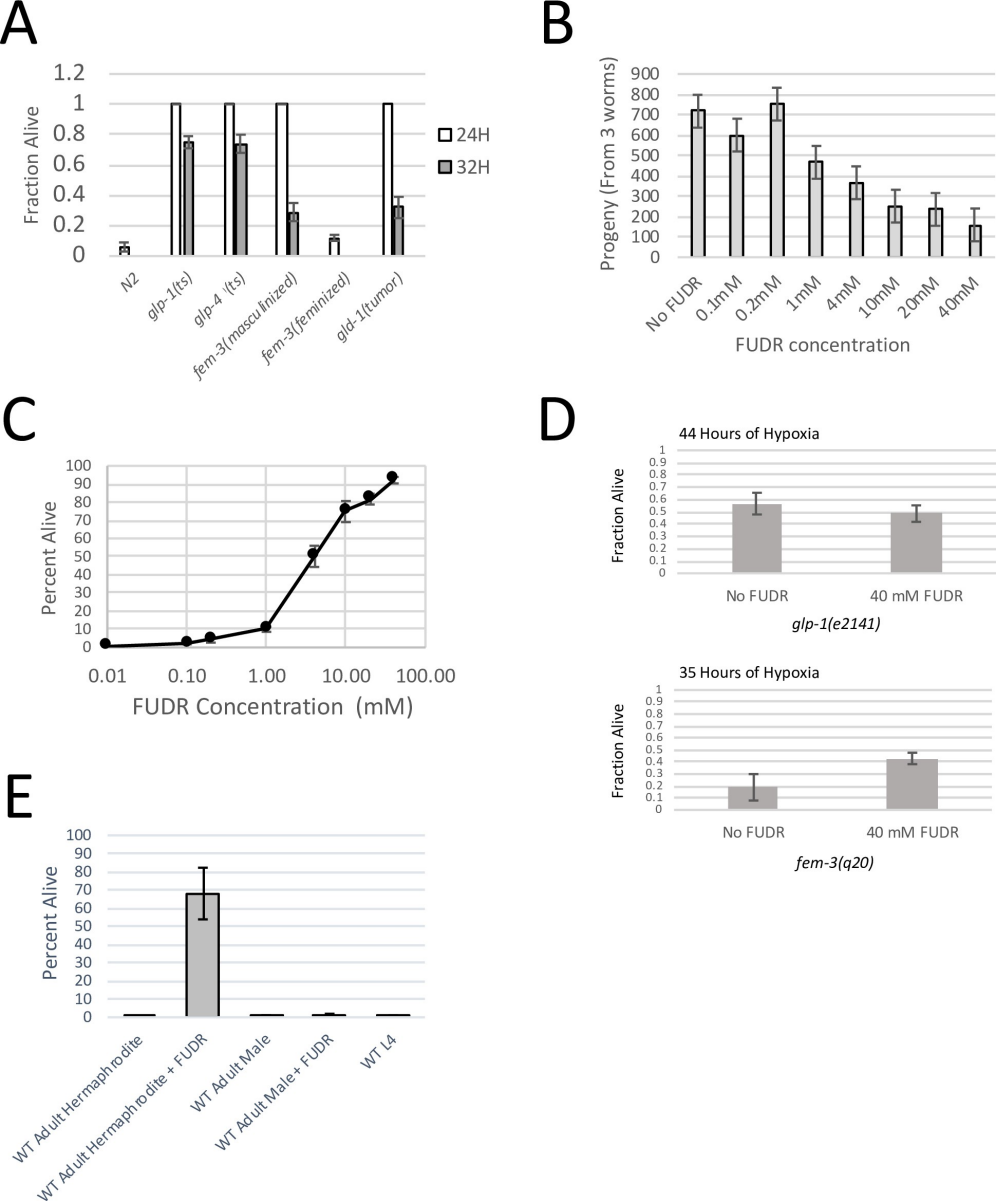
Hypoxia Resistance of Sterile Mutants. (A) The fraction of adult worms surviving either 24 hours or 32 hours of hypoxia at 26.5°C. All animals were grown to adulthood at the non-permissive temperature (25°C) to induce sterility in the germline mutants. Data show that germline ablated animals (*glp-1(ts)* and *glp-4(ts)*), masculinized *fem-3(q20)* mutants (*fem-3(masculinized)*) and *gld-1(tumor)* mutants are highly resistant to hypoxic injury, whereas the feminized *fem-3(e2006)* mutant (*fem-3(feminized)*) displays close to wild type hypoxia sensitivity. Error bars represent standard error. Data are derived from at least 6 biological replicates. (B) The average number of progeny produced by 3 WT worms when treated with different concentrations of FUDR. Data show that the brood size of WT worms decreases as they are exposed to higher concentrations of FUDR. The fertility mid-point is near 4 mM FUDR. Error bars represent standard error. Data are derived from at least 5 biological replicates (C) The percentage of WT worms surviving 24 hours of hypoxia when treated with different concentrations of FUDR. Data show that as FUDR concentration increases, so does hypoxia resistance, mid-point is 4mM FUDR. Error bars represent standard error. Data are derived from at least 5 biological replicates. (D) Fraction of *glp-1(e2141))* and *fem-3(q20)* adults surviving hypoxia with and without 40 mM FUDR. Data show that FUDR increases the survival of *fem-3(q20)* worms, but not the survival of *glp-1(e2141)* adults. 44 hours of hypoxia were used for *glp-1(e2141)* adults and 35 hours of hypoxia were used for *fem-3(q20)* adults. These time points were chosen so that survival was in a range where the impact of FUDR would be visible. Standard error is shown. Data are derived from at least 3 biological replicates. (E) The percentage of WT adult hermaphrodites, WT adult males, and hermaphrodite L4 larvae surviving 24 hours of hypoxia with and without FUDR treatment. WT hermaphrodites are resistant to hypoxia when treated with 40 mM FUDR, whereas WT males +/- FUDR are not resistant to hypoxic injury. WT L4 larvae are also not resistant to hypoxic injury. WT L4 larvae were not treated with FUDR because FUDR is toxic to *C*. *elegans* larvae [23]. Data are derived from at least 4 biological replicates. Error bars represent standard error.

For our hypoxia assay, day 1 adults (12–24 hours after the L4/adult transition) were exposed to hypoxia for 24 or 32 hours, rescued on food containing plates and then scored for survival. Under these conditions, only about 6% of WT adults survive 24 hours of hypoxia and less than 1% survive 32 hours of hypoxia (Fig 1A). Both the *glp-1(e2141)* and *glp-4(bn2)* mutant strains, which do not contain a germline, were highly resistant to hypoxia with >99% of day 1 adults surviving 24 hours of hypoxia and over 70% of day 1 adults surviving 32 hours of hypoxia. We found that the *fem-3(q20)* mutant, which makes sperm but not oocytes, was also resistant to hypoxic injury with >99% of day 1 adults surviving 24 hours of hypoxia and ~30% surviving as much as 32 hours of hypoxia. Finally, we also found that the *gld-1(lf)* tumor strain was resistant to hypoxia, with >99% surviving 24 hours of hypoxia and ~30% surviving 32 hours of hypoxia. In contrast, the *fem-3(e2006)* mutant, which makes oocytes but not sperm, displayed WT levels of hypoxia sensitivity (Fig 1A). Therefore, we found that mutations that prevent oogenesis were also highly resistant to hypoxic injury under our conditions. It should be noted that although four of five strains exhibited strong hypoxia resistance, we found that the germline deficient *glp-1(e2141)* and *glp-4(bn2)* mutants were significantly more resistant to hypoxia than the *fem-3(e2006)* masculinized strain and the *gld-1(lf)* tumor strain, which was most evident at 32 hours of hypoxia (Fig 1A).

### Partial sterility leads to partial hypoxia resistance in WT animals

There are two possible ways that blocking oogenesis could lead to hypoxia resistance. First, hypoxia resistance may only occur if worms are completely prevented from reaching the oogenic stage of development, in which case worms would have to be completely sterile in order to be hypoxia resistant. Alternatively, hypoxia resistance could be proportional to the amount that oogenesis is blocked, in which case a partial reduction in fertility would lead to partial hypoxia resistance. Sterility can be chemically induced in *C*. *elegans* with floxuridine (FUDR). Importantly, reproductive capacity can be titrated by treating worms with different concentrations of FUDR. Therefore, we used a titration of FUDR to induce partial sterility in wild type worms and to determine if partial sterility leads to hypoxia resistance. We found that reproductive output was not reduced at FUDR concentrations below 0.2 mM but was partially suppressed at concentrations ranging from 0.2 mM FUDR to 40 mM FUDR with a midpoint of ~4 mM FUDR (Fig 1B). The sterility brought about by FUDR correlated well with the amount of hypoxia resistance. Specifically, we found that concentrations of 0.2 mM FUDR or less had little impact on hypoxia resistance, whereas concentrations between 0.2 mM FUDR and 40 mM FUDR had an increasing impact on hypoxia resistance with 4 mM FUDR lying at the midpoint of the curve (Fig 1C). Therefore, hypoxia resistance does not require complete sterility and partial sterility leads to partial hypoxia resistance.

We note that FUDR inhibits DNA replication, and therefore could potentially be bringing about hypoxia resistance by acting in somatic tissues, through a distinct mechanism. To address this possibility, we tested FUDR on sterile germline mutants to determine if FUDR was able to induce hypoxia resistance in animals with no germline. If FUDR is impacting hypoxia resistance through the same mechanism as the sterile mutants, we would expect that FUDR would not further increase the hypoxia resistance of sterile mutants. Therefore, we exposed both *glp-1(e2141)* and *fem-3(q20)* animals to FUDR to determine if FUDR could increase the hypoxia resistance of sterile mutants. We found that FUDR did not make *glp-1(e2141)* worms more resistant to hypoxia, but did give a small but significant increase to the hypoxia resistance of the *fem-3(q20)* mutant (Fig 1D). These results imply that inhibition of oogenesis alone is not sufficient to give full hypoxia resistance. Indeed, *fem-3(q20)* animals still undergo germ cell proliferation and differentiation into sperm. Therefore, we suspect that FUDR is still able to act on the partially differentiated germline of the *fem-3(q20)* mutants to bring about additional hypoxia resistance. In contrast, the sterility of the *glp-1(e2141)* mutant is sufficient to get full hypoxia resistance. In this case, FUDR is unable to enhance hypoxia resistance in *glp-1(e2141)* animals because there are no germ cells for FUDR to act on.

### Oogenesis is not required for hypoxia sensitivity

Our results, consistent with previous studies, demonstrate that sterile mutations that prevent oogenesis in adult animals are sufficient to cause hypoxia resistance. This phenomenon suggests that oogenesis may be required for worms to be sensitive to hypoxia. If this hypothesis were true, we would expect that worms that do not make oocytes, such as hermaphrodite L4 larvae and WT males, should also be more resistant to hypoxic injury than WT adults. To address this issue, we subjected WT males and hermaphrodite L4 larvae to 24 hours of hypoxia and scored animals after 24 hours of recovery. We found that neither adult males nor hermaphrodite L4 larvae were more resistant than WT day 1 adults (Fig 1E). These results demonstrate that oogenesis is not a requirement for hypoxia sensitivity at 26.5°C.

We also found that growing WT males on 40 mM FUDR, a concentration that renders hermaphrodites resistant to hypoxia, was not sufficient to make WT males resistant to hypoxic injury (Fig 1E). Therefore, FUDR is only effective at making WT hermaphrodites resistant to hypoxia. Taken together, these results imply that it is the prevention of oogenesis in otherwise oogenic worms that leads to hypoxia resistance, not the lack of oogenesis *per se*.

### Sterility dependent hypoxia resistance only occurs in adults

**Fig 2:**
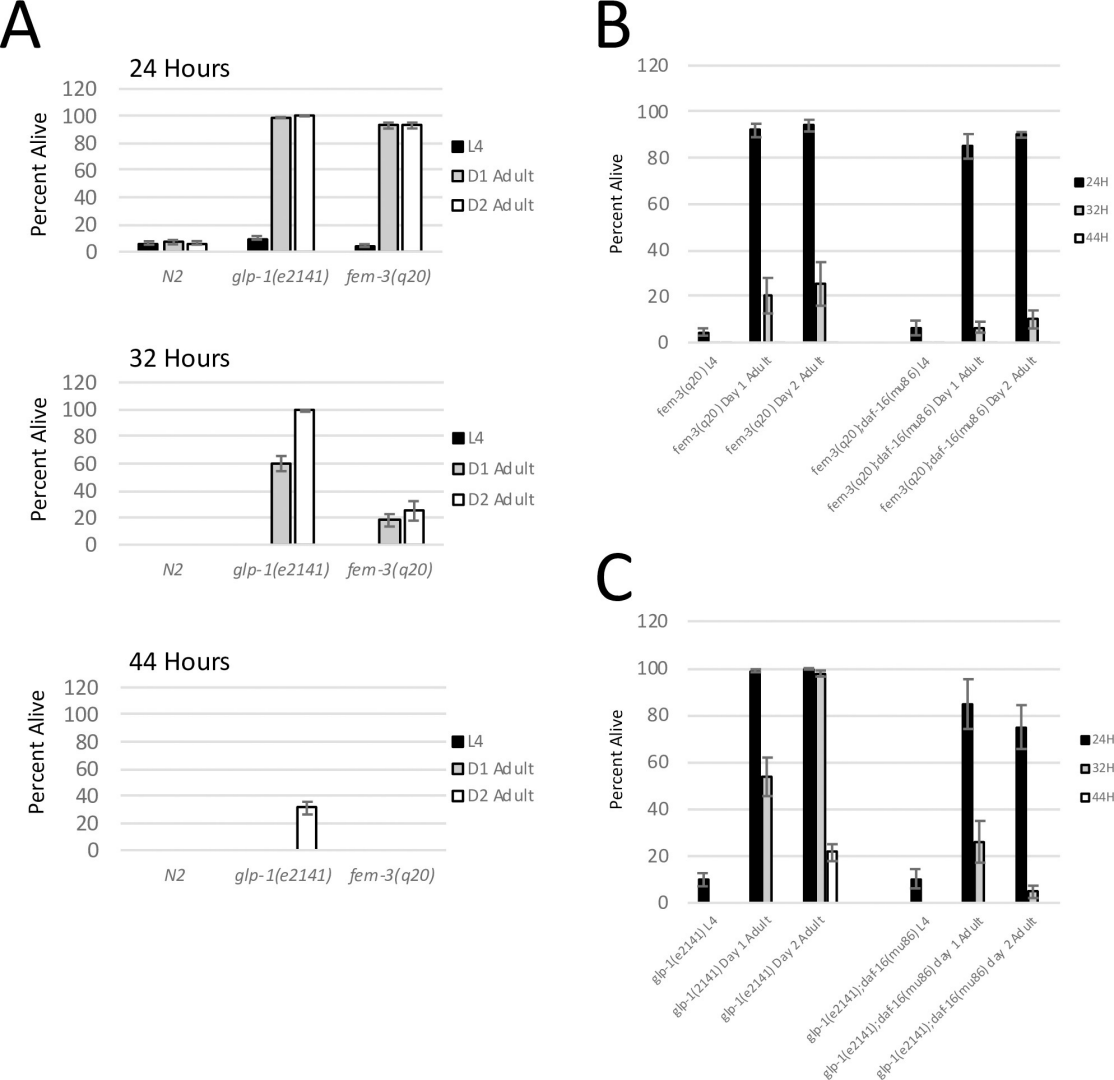
Two Mechanisms of Hypoxia Resistance in Aging Animals. (A) The percentage of worms surviving 24, 32, or 44 hours of hypoxia at 26.5°C. Worms from each genetic background were assayed as L4 larvae, day 1 adults, or day 2 adults. Germline ablated animals (*glp-1(e2141)*) become resistant to hypoxic injury as day 1 adults and even more resistant (super-resistant) as they age to day 2 of adulthood. In contrast, masculinized *fem-3(q20)* mutants become resistant to hypoxic injury as day 1 adults, but do not become super-resistant as they progress to day 2 of adulthood. Error bars represents standard error. Data are derived from at least 6 biological replicates. (B) Percentage of *fem-3(q20)* and *fem-3(q20);daf-16(mu86)* mutants surviving 24, 32, or 44 hours of hypoxia. The graph shows the survival of *fem-3* mutants as L4, day 1 adults, and day 2 adults. Data show that mutation of *daf-16* only partially suppresses the hypoxia resistance of *fem-3(q20)* adults. Error bars represent standard error and data are derived from at least 6 biological replicates (C) Percentage of *glp-1(e2141)* and *daf-16(mu86);glp-1(e2141)* mutants surviving 24, 32 or 44 hours of hypoxia. Data show that primary hypoxia resistance present in day one adults is mostly independent of *daf-16*, whereas the aging-dependent super-resistance of the day 2 *glp-1(e2141)* mutant is completely dependent on *daf-16*. Error bars represent standard error and data are derived from at least 6 biological replicates.

When testing the hypoxia sensitivity of larvae and adults, we observed an interesting phenomenon for the sterile mutants. Specifically, we found that the *glp-1(e2141)* and *fem-3(q20)* mutant strains, which are both very resistant to hypoxia as day 1 adults, were not resistant to hypoxia as L4 larvae (Fig 2A). In a 24-hour exposure to hypoxia, *glp-1(e2141)* and *fem-3(q20)* L4 larvae displayed WT sensitivity to hypoxia, with less than 10% of L4 larvae from both strains surviving 24 hours of hypoxia. In contrast, 100% of *glp-1(e2141)* day 1 adults and >95% of *fem-3(q20)* day 1 adults survived the same 24-hour exposure to hypoxia. The difference between L4 larvae and day 1 adults was even more apparent at longer hypoxia exposures. Indeed, less than 1% of the L4 larvae from both sterile mutants survived 32 hours of hypoxia, whereas ~60% of *glp-1(e2141)* day 1 adults and ~20% of *fem-3(q20)* day 1 adults survived the same 32-hour exposure (Fig 2A). Therefore, in just the time it takes to develop from L4 larvae to young adult (~12 hours), the *glp-1(e2141)* and *fem-3(q20)* mutants transition from hypoxia sensitive L4 larvae to robustly hypoxia resistant day 1 adults. Such a transition is not observed in WT animals, however, as WT L4 larvae and day 1 adults displayed similar levels of hypoxia sensitivity after 24 hours of hypoxia exposure (Fig 2A). We note that these results are further evidence that oogenesis is not required for hypoxia sensitivity, as *glp-1(e2141)* and *fem-3(q20)* L4 larvae do not undergo oogenesis, yet they still display wild-type sensitivity to hypoxic injury.

### *Glp-1(e2141)* mutants become “super-resistant” to hypoxia as they age

When testing the impact of developmental stage on hypoxia resistance, we also found that the hypoxia resistance of germline ablated animals increased significantly as adults progressed to day 2 of adulthood (36–48 hours after the L4/adult transition). Indeed, >99% of *glp-1(e2141)* day 2 adults survived 32 hours of hypoxia compared to only ~60% of day 1 adults (Fig 2A). Remarkably, ~30% of day 2 adults survived as much as 44 hours of hypoxia, compared to less than 1% of *glp-1(e2141)* day 1 adults. Therefore, for the *glp-1(e2141)* mutant, L4 larvae display wild-type sensitivity to hypoxia, day 1 adults display strong resistance to hypoxia, and day 2 adults display even stronger resistance to hypoxia. We have called this aging dependent increase in hypoxia resistance “super-resistance”, as this is the strongest hypoxia resistance we have observed.

The aging dependent super resistance phenomenon was limited to the *glp-1(e2141)* mutant, however, as the *fem-3(q20*) mutant displayed a significant increase in hypoxia resistance between L4 and day 1 of adulthood, but saw no further increase in hypoxia resistance as they aged to day 2 of adulthood (Fig 2A). Taken together, these results suggest that there are multiple mechanisms of hypoxia resistance in sterile animals. A mechanism of primary hypoxia resistance which occurs in animals with inhibited oogenesis between L4 and day 1 of adulthood, and a second mechanism of “super-resistance” which we have only observed in germline deficient mutants as they progress to day 2 of adulthood. Hypoxia resistance did not further increase as animals aged past day 2 of adulthood (S1 Fig).

### Super resistance of the *glp-1(e2141)* mutant is dependent on insulin signaling

The insulin signaling pathway, operating through the DAF-16/FoxO transcription factor, is involved in a wide range of aging and stress related phenomena. Indeed, it is established that the longevity of the *glp-1(e2141)* mutant is dependent upon *daf-16* [16]. To determine if the hypoxia resistance of sterile mutants is also dependent upon *daf-16*, we acquired a *daf-16(mu86);glp-1(e2141)* double mutant strain and we built a *fem-3(q20);daf-16(mu86)* double mutant strain. We found that mutation of *daf-16* only mildly suppressed the hypoxia resistance of *glp-1(e2141)* and *fem-3(q20)* day 1 adults (Fig 2B and 2C). In contrast, we found that the aging dependent increase in hypoxia resistance in the *glp-1(e2141)* strain was completely suppressed by *daf-16* mutation. Specifically, day 2 *glp-1(e2141)* adults were considerably more resistant to hypoxia than day 1 adults, but day 2 *daf-16(mu86);glp-1(e2141)* double mutants were no more resistant to hypoxic injury than day 1 adults (Fig 2C).

These results further solidify the notion that two distinct mechanisms of hypoxia resistance are operating in sterile mutants. First, a *daf-16 independent* mechanism that gives hypoxia resistance to both *fem-3(q20)* and *glp-1(e2141*) mutants after the transition from L4 larvae to day 1 of adulthood, this mechanism is consistent with what has previously been reported [10]. Second, a *daf-16 dependent* mechanism that provides super-resistance to *glp-1(e2141)* mutants when they reach day 2 of adulthood. The *daf-16* dependent super resistance mechanism operates in the *glp-1(e2141)* mutant as it ages to day 2 of adulthood, but not in the masculinized *fem-3(q20)* mutant, and has not previously been reported.

### Germline mutations prevent hypoxia dependent mitochondrial protein aggregation

Previous studies have shown that mitochondrial protein aggregation is one of the physiological hallmarks of hypoxic injury in *C*. *elegans* [17,18]. Mitochondrial protein aggregation has been observed using transmission electron microscopy or by using a GFP reporter strain that expresses the mitochondrial UCR-11 protein (ubiquinol-cytochrome C reductase) fused to GFP in the body wall muscle. Mitochondrial protein aggregation is often accompanied by a swelling/rounding of body wall muscle mitochondria. Both mitochondrial protein aggregation and swelling are reversible under sublethal conditions, as mitochondria return to normal shape and aggregation is reversed 24 hours after return to normal oxygen conditions [17]. Although previous studies have shown a correlation between mitochondrial protein aggregation and hypoxia sensitivity, it is not yet known if mitochondrial protein aggregates cause hypoxic injury and whether or not hypoxia resistant mutants prevent the formation of protein aggregates.

**Fig 3:**
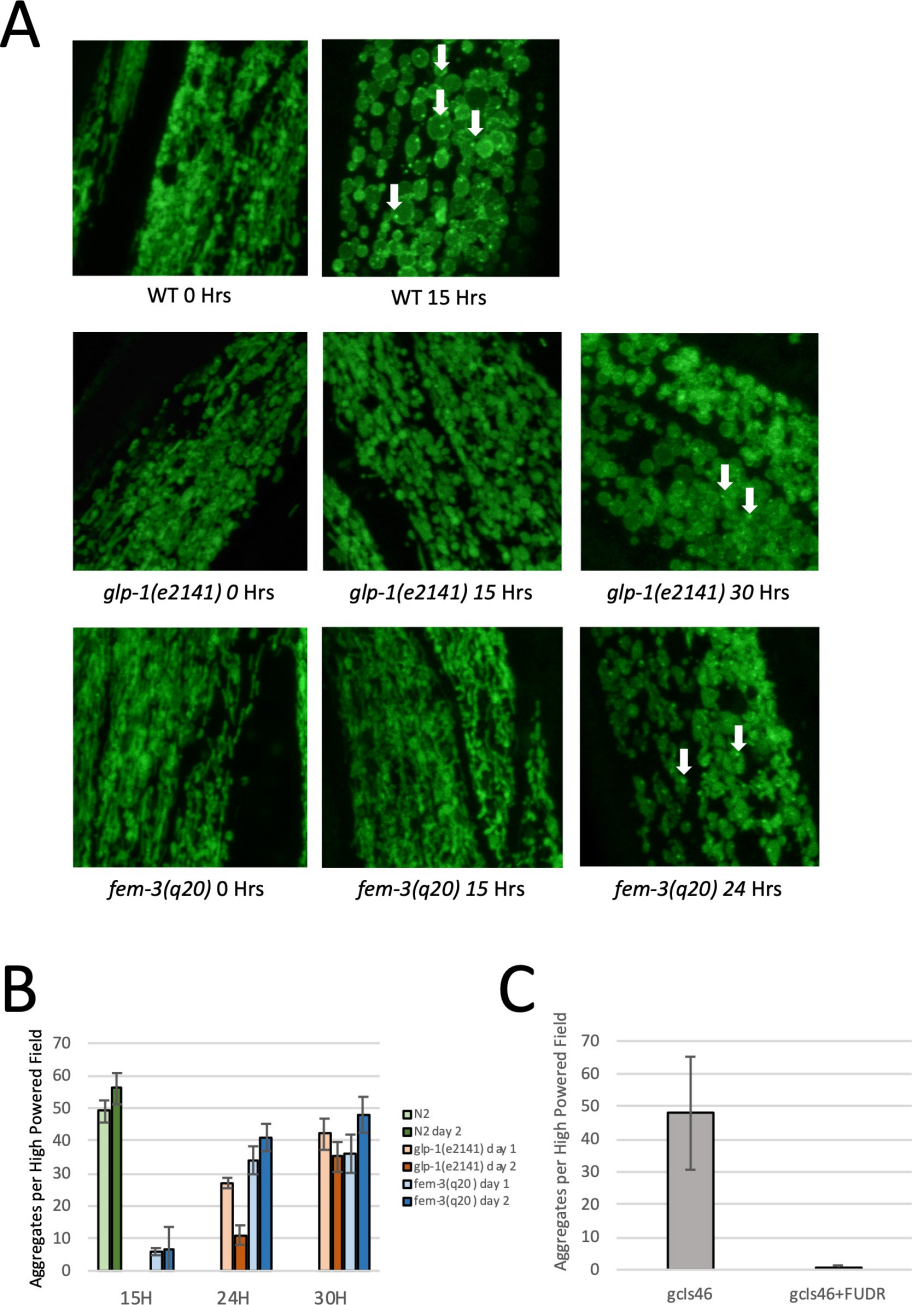
Sterile Mutations Prevent Hypoxia Dependent Mitochondrial Protein Aggregation. (A) GFP fluorescence images of WT, *glp-1(e2141)* and *fem-3(q20)* day 1 adults. Fluorescence is from a GFP tagged UCR-11 protein which localizes to body wall muscle mitochondria. The identity of the strain background being shown, along with the time of hypoxia exposure is written below each image. Examples of mitochondrial protein aggregates are indicated by the white arrows. 15 hours of hypoxia is sufficient to cause rounding of the mitochondria and significant mitochondrial protein aggregation in WT worms, but not in *glp-1(e2141)* or *fem-3(q20)* mutants. 24 and 30 hours of hypoxia are sufficient to cause mitochondrial rounding and protein aggregation in *fem-3(q20)* and *glp-1(e2141)* adults respectively. (B) Quantification of protein aggregation after hypoxia. The average number of aggregates observed per high-powered field is displayed. The quantity of aggregates is shown after 15, 24, or 30 hours of hypoxia at 26.5°C. N/A is displayed for WT worms at 24 and 30 hours because WT worms do not survive these longer hypoxia exposures. Error bars represent standard error. Data are derived from at least 8 worms. (C) Quantification of protein aggregates in WT worms after 15 hours of hypoxia at 26.5°C with and without FUDR. Data show that FUDR nearly eliminates mitochondrial protein aggregation in WT worms after hypoxia exposure. Error bars represent standard error. Data represent at least 4 biological replicates.

To examine mitochondrial protein aggregation in germline mutants, we crossed a *ucr-11*::*gfp* reporter strain into the *glp-1(e2141)* and *fem-3(q20)* mutant backgrounds. We then observed hypoxia dependent mitochondrial protein aggregation in both mutant reporter strains and compared them to the WT reporter strain. To account for the super-resistance mechanism, we examined both day 1 and day 2 adults of each strain. For WT animals, we found that 15 hours of hypoxia was sufficient to cause rounding/swelling of mitochondria along with the formation of abundant protein aggregates (Fig 3A). Quantification of protein aggregates in WT animals revealed nearly 50 aggregates per high powered field (HPF) after 15 hours of hypoxia (Fig 3B). We observed no differences between day 1 and day 2 WT worms. In contrast, 15 hours of hypoxia led to no mitochondrial swelling or protein aggregation and less than 5 aggregates/HPF in the *fem-3(q20)* mutant and no swelling or detectable aggregates in the *glp-1(e2141)* background (Fig 3A and 3B). For both germline mutants, longer periods of hypoxia exposure were required to cause significant mitochondrial protein aggregation. For *fem-3(q20)* animals and *glp-1(e2141)* day 1 adults, 24 hours of hypoxia were sufficient to cause mitochondrial swelling and significant protein aggregation, and for *glp-1(e2141)* day 2 adults, 30 hours of hypoxia were required to cause swelling and significant mitochondrial protein aggregation (Fig 3A and 3B). WT animals did not survive either a 24-hour or 30-hour hypoxia exposure. The fact that day 2 *glp-1(e2141)* animals are more resistant to mitochondrial protein aggregation than day 1 *glp-1(e2141)* animals implies that both the primary sterility dependent mechanism and the super-resistance mechanism protect against mitochondrial protein aggregation.

We also used FUDR to determine if chemically induced sterility also prevented mitochondrial protein aggregation. WT animals carrying the *ucr-11*::*gfp* reporter were treated with 40 mM FUDR and then subjected to 15 hours of hypoxia. 15 hours of hypoxia were sufficient to induce significant protein aggregation in untreated animals, whereas animals treated with 40 mM FUDR displayed very little protein aggregation (Fig 3C). Taken together, these results show that either sterile mutations or compounds that cause sterility are sufficient to prevent hypoxic injury and reduce the amount of hypoxia induced mitochondrial protein aggregation.

### RNAseq analysis of development and aging in germline mutants

The most evident strategy for identifying changes in gene expression associated with hypoxia resistance would be to compare hypoxia sensitive WT animals to hypoxia resistant sterile mutants. There are significant disadvantages to making such comparisons, however, because WT worms possess an entire germline along with all of its associated gene expression, whereas *glp-1(e2141)* animals possess no germline expression at all. Therefore, it is to be expected that there would be massive differences in gene expression solely due to the presence or absence of a germline, which would make it exceedingly difficult to isolate gene expression changes specifically associated with hypoxia resistance. Our findings gave us a much more attractive alternative for gene expression analysis. Indeed, in just a 12-hour period, *glp-1(e2141)* animals transition from hypoxia sensitive L4 larvae into highly resistant adults, and then in another 24 hours *glp-1(e2141)* animals become super-resistant to hypoxia. Thus, gene expression can be compared in the same germline deficient genetic background at three different time points, eliminating complications associated with gene expression in the germline. For these reasons, we carried out RNAseq analysis to examine changes in gene expression that occurred in *glp-1(e2141)* animals between the hypoxia sensitive L4 larval stage of development, the hypoxia resistant day 1 of adulthood, and the hypoxia super-resistant day 3 of adulthood.

As would be expected from any gene expression analysis, we observed many genes both induced and repressed in *glp-1(e2141)* animals as they progressed from L4 to day 1 and then to day 3 of adulthood (S1 Table). The most strongly regulated genes were involved in molting, which would be expected in animals transitioning through the last larval molt. While there were many other genes both activated and repressed in *glp-1(e2141)* adults, we decided to focus our study on the genes that stood out from our RNAseq data as the most likely to be involved in hypoxia resistance. In this regard, we focused our attention on genes involved in protein translation, particularly cytosolic and mitochondrial ribosomal protein genes (cRPGs and mRPGs), which were broadly repressed in *glp-1(e2141)* day 1 adults and further repressed in day 3 adults (S2 Fig). Specifically, 16 out of 78 cytosolic ribosomal protein genes and 57 out of 71 mitochondrial ribosomal protein genes were repressed by greater than 2-fold in day 1 adults (S2 and S3 Tables). Repression was even stronger in day 3 adults where 73 out of 78 cRPGs and 69 out of 71 mRPGs were repressed by greater than two-fold. Since there is no rule that a gene has to be repressed by greater than 2-fold to be biologically significant, we examined the repression of all cRPGs and mRPGs, including genes that were repressed by less than the 2-fold cutoff (S2 and S3 Tables). Importantly, we found that all cRPGs and mRPGs were repressed in day 1 and day 3 adults and appeared to be repressed together as groups. In other words, although only 16 out of 78 cRPGs were repressed more than 2-fold in day 1 adults, the remaining 62 cRPGs were still repressed by close to 2-fold, nearly all ranging from 1.6-fold to 2.0-fold (mean = 1.8-fold). Treating all of the cRPGs together as a group makes our analysis more statistically robust. For example, if one gene is repressed by 1.8-fold, the statistical significance may not be very high, but if *all* 78 cRPGs are repressed to a similar degree with an average of 1.8-fold, 1.8-fold becomes highly significant (P-value < 0.00001 when compared to background genes).

**Fig 4:**
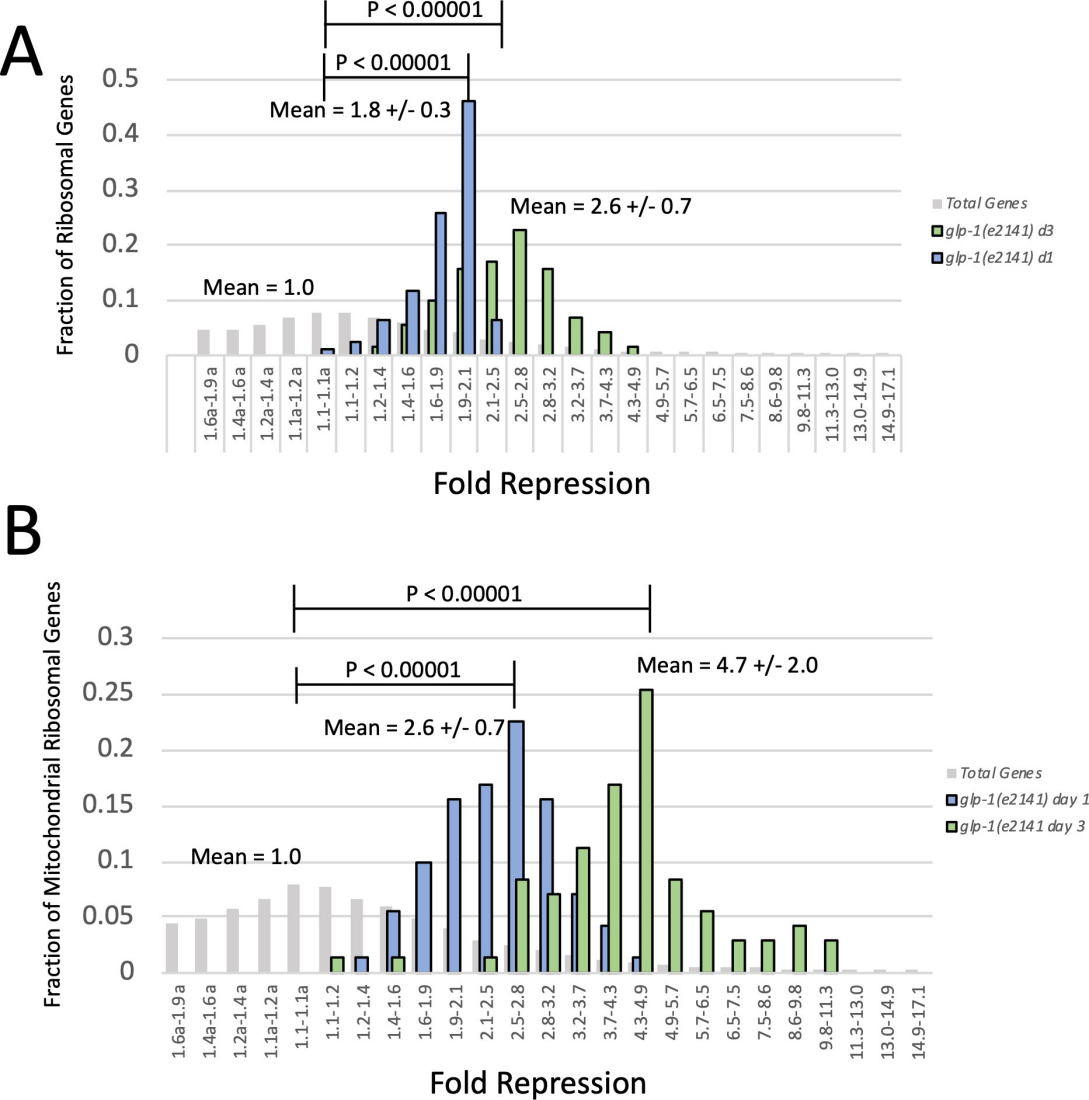
Ribosomal Protein Genes (RPGs) are Repressed in *glp-1(e2141)* Adults. (A) Histograms of cytosolic ribosomal protein genes (cRPGs) binned by fold repression/activation. Activation is indicated on the x-axis by an (a) next to the bin range. Data represent the fraction of total cytosolic ribosomal protein genes residing in each range of repression. Blue bars represent repression between L4 and day 1 of adulthood and green bars represent repression between L4 and day 3 of adulthood. Gray bars represent the fraction of total genes in each range of repression or induction. Mean is the average repression of all cRPGs at day 1 or day 3 of adulthood along with the standard deviation. Data show that cRPGs are repressed together (as indicated by tight distributions) in day 1 adults by an average of 1.8-fold and further repressed together in day 3 adults by an average of 2.6-fold. The repression of cRPGS at both day 1 and day 3 of adulthoods is highly significantly in comparison to total genes (P value < 0.00001). (B) Histograms of mitochondrial ribosomal protein genes (mRPGs) binned by fold repression/activation. Blue bars represent the repression of mitochondrial ribosomal protein genes between L4 and Day 1 of adulthood, and green bars represent the repression of mRPGs between L4 and day 3 of adulthood. Gray bars represent the fraction of total genes in each range of repression or induction. Mean is the average repression of all mRPGs at day 1 or day 3 of adulthood along with the standard deviation. Data show that mRPGs are repressed together in day 1 adults by an average of 2.6-fold and further repressed together in day 3 adults by an average of 4.7-fold. Repression of mRPGs is significantly stronger than cRPGs (P-value < 0.00001). The repression of mRPGs at both day 1 and day 3 of adulthoods is highly significantly in comparison to total genes (P value < 0.00001). RNAseq data were derived from 5 biological replicates. Statistics were calculated using a T-test for two independent means. E.g., the mean of cRPGs vs the mean of total genes.

To more clearly represent the coordinated regulation of cRPGs and mRPGs, we plotted histograms representing the fraction of total cRPGs and mRPGs in different ranges of repression. As would be expected if the different RPG groups were regulated together, cRPGs and mRPGs were not randomly distributed, but rather formed tight distributions. When plotted as a histogram, cRPGs formed a tight distribution with a mean of 1.8-fold repression in day 1 adults (Fig 4A). This level of repression was highly significant in comparison to background (P-value < 0.00001). Repression of cRPGs was even stronger in day 3 adults, where cytosolic ribosomal protein genes formed a tight distribution with a mean of 2.6-fold repression, also highly significant in comparison to background (P-value < 0.00001) (Fig 4A). Mitochondrial ribosomal protein genes were also regulated together and were repressed more robustly than the cRPGs (Fig 4B). Indeed, when plotted as histograms based on the degree of repression, mRPGs formed distinct distributions with a mean of 2.6-fold repression in day 1 adults and a mean of 4.7-fold repression in day 3 adults. All of these distributions are significantly different (P-value < 0.00001) than the distribution of total *C*. *elegans* genes, which is centered at 1.0-fold repression/activation (Fig 4). Therefore, it is clear that both cRPGs and mRPGs are regulated together as groups and are significantly repressed in the *glp-1(e2141)* mutant as it develops from L4 to adulthood, and further repressed as they progress past day 1 of adulthood (Table 1).

**Table 1:** Repression of Translation Genes in D1 and D3 Adults.

	*glp-1(e2141)*		*fem-3(q20)*	
	L4 > d1	L4 > d3	L4 > d1	L4 > d3
Cytosolic Ribosomal Genes	1.8 +/- 0.3	2.6 +/-0.8	1.6 +/- 0.2	1.2 +/- 0.2
tRNA Synthetase Genes	2.0 +/- 0.5	1.9 +/- 0.5	1.3 +/- 0.2	1.1 +/- 0.4
Nucleolar Genes	4.3 +/- 2.1	3.3 +/- 1.2	1.5 +/- 0.5	1.1 +/- 0.3
Mitochondrial Ribosomal Genes	2.6 +/- 0.7	4.7 +/- 1.9	1.8 +/- 0.5	1.5 +/- 0.5
Mitochondrial Translocases	2.3 +/- 0.4	4.0 +/- 0.6	1.5 +/- 0.2	1.0 +/- 0.3
Mitochondrial tRNA Synthetase Genes	1.9 +/- 0.9	2.9 +/- 1.4	1.0 +/- 0.2	1.1 +/- 0.3

### Other translation genes are repressed in *glp-1(e2141)* adults

In addition to the ribosomal protein genes, other genes involved in protein translation were repressed in *glp-1(e2141)* adults. In fact, tRNA synthetase genes also appeared to be regulated together as a group. For example, 19 out of 20 cytosolic tRNA synthetase genes were repressed in day 1 adults and nearly half were repressed by more than 2-fold (mean = 2.0-fold) (Tables 1 and S4). Unlike ribosomal protein genes, cytosolic tRNA synthetase genes were not further repressed in day 3 adults (mean = 1.9-fold repression). Mitochondrial tRNA synthetase genes were repressed by an average 1.9-fold in day 1 adults and more significantly repressed in day 3 adults (mean = 2.9-fold). We also identified eleven genes involved in nucleolar function, including a regulator of nucleolar function, the *fib-1* gene, as repressed in day 1 adults but not further repressed in day 3 adults (Tables 1 and S4 Table).

Taken together, we have identified three classes of gene regulation for genes involved in protein translation (Table 1). First, the ribosomal genes, which are repressed ~1.8-fold in day 1 adults and further repressed by ~2.6-fold in day 3 adults. Second, mitochondrial ribosomal protein genes, which are repressed more strongly at ~2.6-fold in day 1 adults and further repressed by ~4.7-fold in day 3 adults. Third, cytosolic tRNA synthetases and nucleolar genes, which are repressed in day 1 adults, but *not further repressed* in day 3 adults.

### Mitochondrial protein translocation genes are co-regulated with ribosomal genes

Our results show that genes that function together in translation are regulated together in developing and aging *glp-1(e2141)* mutants. Thus, we asked if any other gene families were regulated in a similar fashion to the translation genes, as these genes may also be functionally related to translation. Indeed, we found that 12 genes involved in mitochondrial protein *translocation* were repressed to a similar degree as mitochondrial ribosomal genes (Tables 1 and S4 Table). Specifically, translocation genes were repressed with a mean of 2.3-fold in day 1 adults and 3.9-fold in day 3 adults. This level of repression closely mirrors the level of repression of mitochondrial ribosomal protein genes, which were repressed by an average of 2.6-fold in day 1 adults and 4.7-fold in day 3 adults (Table 1). Therefore, it appears that the mitochondrial translocase genes are co-regulated with genes involved in mitochondrial protein translation, suggesting that that mitochondrial protein *translocation* is functionally linked to mitochondrial protein *translation* in some fashion that requires them to be transcriptionally co-regulated.

### Regulation of translation genes in *fem-3(q20)* adults

**Fig 5:**
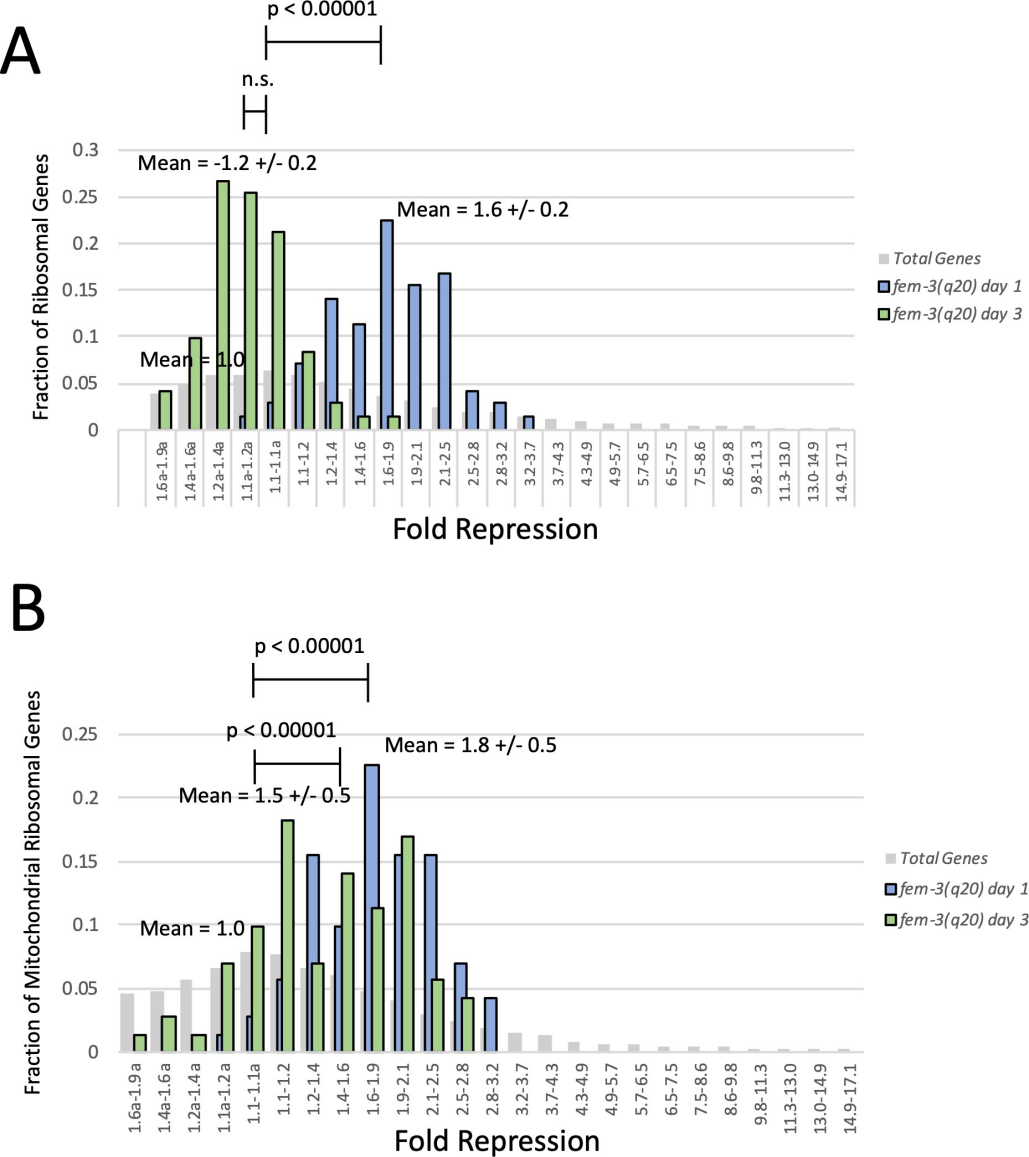
Repression of Ribosomal Protein Genes in *fem-3(q20)* Mutants. (A) Histograms of cytosolic ribosomal protein genes in a *fem-3(q20)* mutant binned by fold repression/activation. Data represent the fraction of cRPGs residing in each range of repression. Blue bars represent repression between L4 and day 1 of adulthood and green bars represent repression between L4 and day 3 of adulthood. Means represent the average repression of all ribosomal protein genes in day 1 or day 3 adults and are shown along with standard deviations. Data show that cRPGs are mildly repressed by 1.6-fold between L4 and day 1 of adulthood (P value < 0.00001). In contrast to *glp-1(e2141)* animals, however, cRPGs are *not* further repressed in the *fem-3(q20)* day 3 adults. In fact, between L4 and day 3 of adulthood cRPGs are not significantly repressed at all in the *fem-3(q20)* mutant. (B) Histograms of mitochondrial ribosomal protein genes (mRPGs) binned by fold repression. Data represent the fraction of all mRPGs residing in each range of repression. Blue bars represent repression between L4 and day 1 of adulthood and green bars represent repression between L4 and day 3 of adulthood. Data show that mRPGs are mildly repressed in day 1 adults (mean = 1.8-fold), but not further repressed in day 3 adults (mean = 1.5-fold). Repression in both day 1 and day 3 adults is highly significant compared to total genes (P values < 0.00001). Data are derived from 3 biological replicates. Statistics were calculated using a T-test for two independent means. E.g., the mean of all cRPGs vs the mean of total genes.

If reduced expression of translation genes was responsible for the primary hypoxia resistance mechanism observed in both *glp-1(e2141)* and *fem-3(q20)* mutants, then we would expect that translation genes would also be repressed in the *fem-3(q20)* mutant during the L4 to adult transition, but not further repressed as *fem-3(q20)* animals progressed to day 3 of adulthood. For this reason, we used RNAseq to identify changes in gene expression that occur in the *fem-3(q20)* mutant between L4 and day 1 and day 3 of adulthood (S2 Fig and S1 Table). Our analysis revealed that both cytosolic and mitochondrial ribosomal protein genes were repressed together in *fem-3(q20)* day 1 adults (S2 Fig and S2 and S3 Tables), just not as strongly as that observed in *glp-1(e2141)* adults. Plotting RPGs as histograms revealed that cRPGs were repressed in day 1 adults with a mean of 1.6-fold repression and mRPGs were repressed in day 1 adults with a mean of 1.8-fold repression (Fig 5A and 5B). For both cytosolic and mitochondrial ribosomal protein genes this degree of repression is lower than that observed in *glp-1(e2141)* animals (P-value <0.00001), however repression of both groups of RPGs in day 1 *fem-3(q20)* adults is statistically significant when compared to total genes (P-values < 0.00001). In contrast to the *glp-1(e2141)* mutant, both cytosolic and mitochondrial ribosomal protein genes were *not* further repressed in day 3 animals (Fig 5A and 5B and Table 1). In fact, cRPGs were not at all repressed in day 3 animals and mRPGs were also repressed less in day 3 animals than day 1 animals. Therefore, protein translation genes are indeed repressed in day 1 *fem-3(q20)* adults, albeit to a lesser degree than in *glp-1(e2141)* animals, but ribosomal protein genes were not further repressed in day 3 adults. We note that this pattern corresponds to hypoxia resistance in germline mutants, which occurs between L4 and day 1 of adulthood in the *fem-3(q20)* mutant, but does not become stronger after day 1 of adulthood in the *fem-3(q20)* mutant. Additionally, the fact that repression of RPGs is weaker in *fem-3(q20)* animals, when compared to the *glp-1(e2141)* mutant, is consistent with the observation that hypoxia resistance is lower in *fem-3(q20)* day 1 adults. Other translation genes were repressed to a lower degree in *fem-3(q20)* day 1 adults, and were not further repressed in day 3 adults (Table 1 and S4).

### Mutation of *daf-16* specifically suppresses repression of cytosolic ribosomal protein genes

Our RNAseq data gave us a potential explanation for the hypoxia resistance observed in day 1 adults and the hypoxia super-resistance observed in day 3 adults. Because mutation of *daf-16* specifically suppresses the hypoxia super resistance that occurs in day 3 adults, we next used RNAseq to examine the expression of ribosomal protein genes in a *daf-16(mu86);glp-1(e2141)* double mutant to determine if mutation of *daf-16* impacted the developmental repression of cRPGs and mRPGs between L4 and day 3 of adulthood (S1 Table).

**Fig 6:**
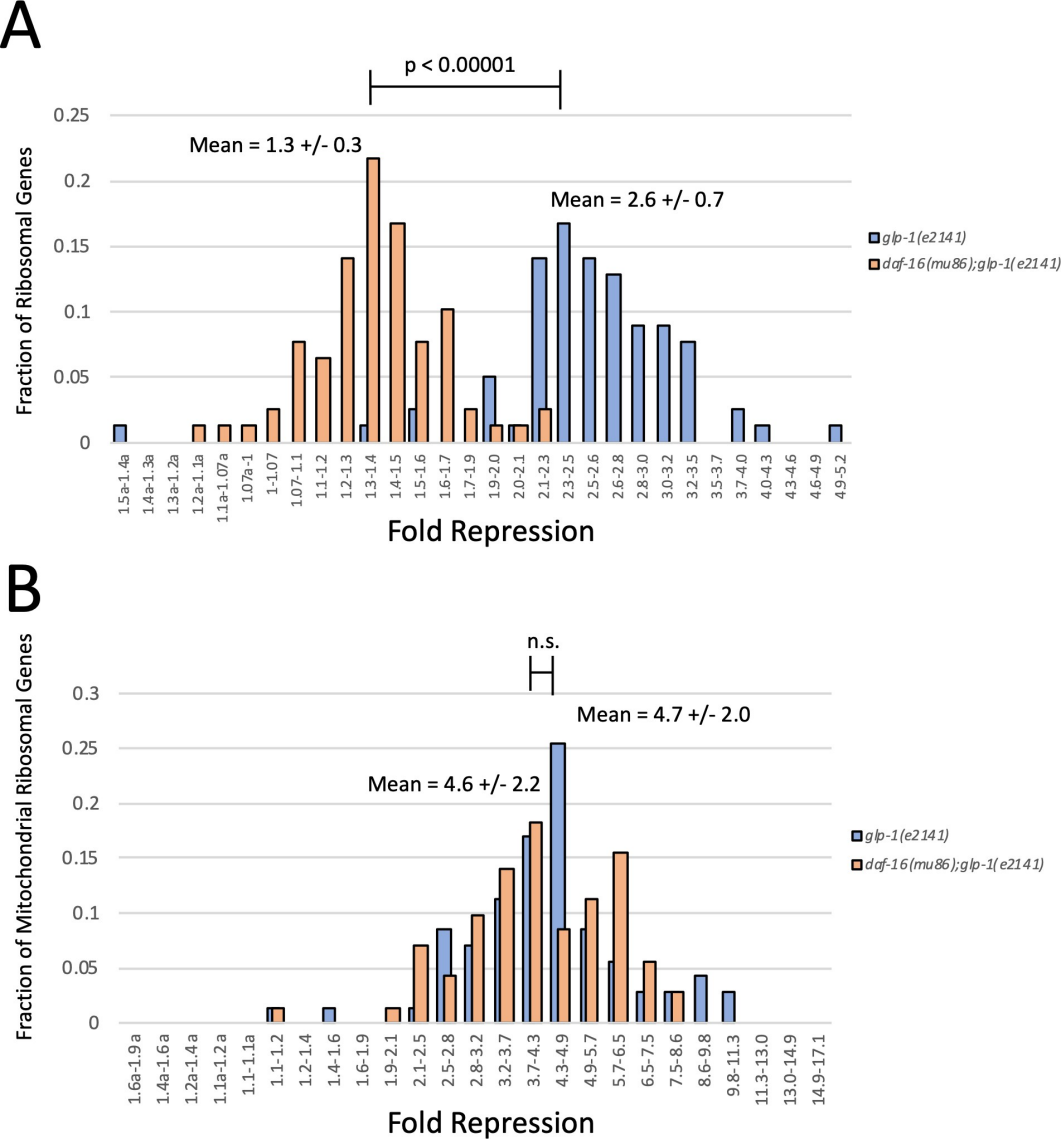
Mutation of *daf-16* Specifically Suppresses the Repression of cRPGs in Day 3 Adults. Histograms of cytosolic ribosomal protein genes binned by fold repression between L4 and day 3 of adulthood. Data represent the fraction of cRPGs residing in each range of repression. Blue bars represent cRPGs from a *glp-1(e2141)* mutant and orange bars represent cRPGs from a *daf-16(mu86);glp-1(e2141)* double mutant. Data show that cRPGs are significantly repressed (mean = 2.6-fold) in *glp-1(e2141)* animals, however repression of cRPGs in the *glp-1(e2141)* mutant is nearly eliminated by mutation of *daf-16* (P value < 0.00001). (B) Histograms of mitochondrial ribosomal protein genes binned by fold repression in day 3 adults. Blue bars represent mRPGs from a *glp-1(e2141)* mutant and orange bars represent mRPGs from a *daf-16(mu86);glp-1(e2141)* double mutant. Data show that mRPGs are repressed in the *glp-1(e2141)* mutant (mean = 4.7-fold). In contrast to cRPGs, however, repression of mRPGs in day 3 animals is not impacted by mutation of *daf-16*. RNAseq data are derived from 4 replicates of the *daf-16(mu86);glp-1(e2141)* double mutant and 5 replicates of the *glp-1(e2141)* mutant. Statistics were calculated using a T-test for two independent means. E.g., the mean of cRPGs vs the mean of total genes.

We found that mutation of *daf-16* specifically influenced the repression of cytosolic ribosomal protein genes (Figs 6 and S2 and S2 and S3 Tables). In day 3 *glp-1(e2141)* animals, 72 out of 78 cytosolic ribosomal genes were repressed greater than 2-fold. Plotting them as a histogram, they form a tight distribution with a mean of 2.6-fold repression (Fig 6A). In a *daf-16(mu86);glp-1(e2141)* mutant, however, only 3 of 78 cytosolic ribosomal genes are repressed greater than 2-fold. When plotted as a histogram, cRPGs form a tight distribution with an average of only 1.3-fold repression in *daf-16(mu86);glp-1(e2141)* day 3 adults (Fig 6A).

Remarkably, mutation of *daf-16* only impacted the expression of genes involved in cytosolic protein translation (S2 Fig and S2–S4 Tables). Repression of cytosolic tRNA synthetases was partially suppressed, but mitochondrial ribosomal protein genes, mitochondrial tRNA synthetases, and mitochondrial translocases were repressed to the same degree that was observed in the *glp-1(e2141)* single mutant. These results demonstrate that mutation of *daf-16* specifically suppresses the repression of cytosolic ribosomal protein genes, arguing that specific repression of cRPGs by *daf-16* is likely the key factor in hypoxia super-resistance.

### Genes involved in oxidative phosphorylation are repressed in day 3 adults

**Fig 7:**
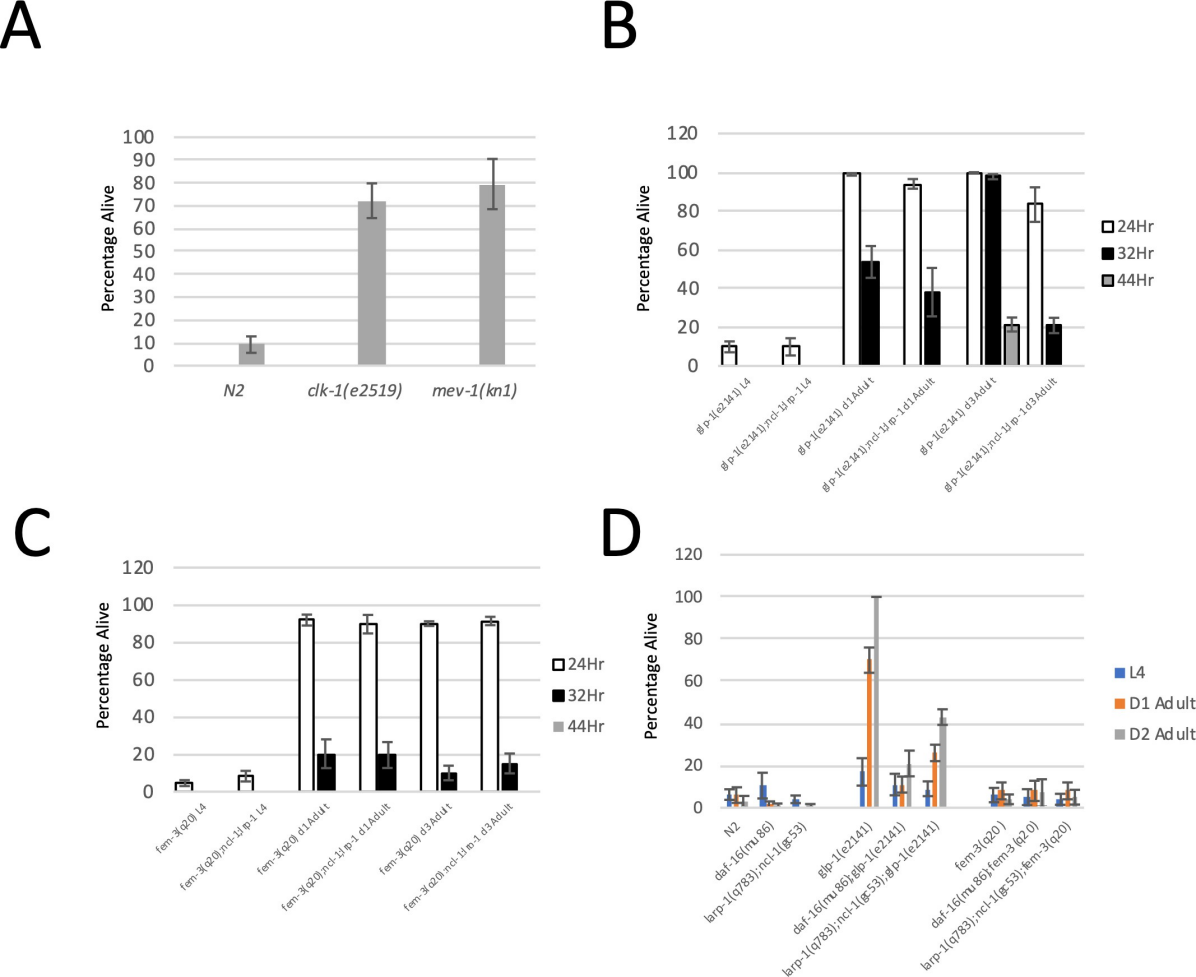
Potential Mechanisms of *glp-1(e2141)* Super Resistance. (A) Hypoxia resistance of oxidative phosphorylation mutants. Data show that both *clk-1* and *mev-1* mutants are highly resistant to hypoxic injury. Data are derived from at least 4 biological replicates. (B) Percentage of *glp-1* mutant worms surviving 24, 32, and 44 hours of hypoxia. Mutation of *ncl-1* and l*arp-1* together does not suppress the day 1 adult hypoxia resistance of *glp-1(e2141)* mutants, but completely suppresses the aging dependent super resistance of day 2 *glp-1(e2141)* mutants. (C) Percentage of worms surviving 24, 32, and 44 hours of hypoxia. *Fem-3(q20)* mutants become more resistant to hypoxia on day 1 of adulthood, but do not become super resistant by day 2 of adulthood. Mutation of *ncl-1* and *larp-1* together does not suppress the hypoxia resistance of day 1 or day 3 *fem-3(q20)* adults. Data represent 6 biological replicates. (D) Percentage of worms surviving heat stress (10.5 hours at 35°C). *glp-1(e2141)* day 1 adults survive 10.5 hours of heat stress, whereas L4 worms do not. Mutation of *daf-16* suppresses the heat resistance of *glp-1(e2141)* worms. Neither *fem-3(q20)* adults or L4 larvae survive 10.5 hours of heat stress. Data represent 4 biological replicates.

In addition to genes involved in protein translation, we also observed that genes involved in oxidative phosphorylation were repressed between Day 1 of adulthood and Day 3 of adulthood (S3 Fig and S1 Table). Thus, repression of oxidative phosphorylation could also be a mechanism of the increased hypoxia resistance observed after day 1 of adulthood. To determine if reduced oxidative phosphorylation can lead to hypoxia resistance, we obtained two mutants in the oxidative phosphorylation mechanism, *clk-1(e2519)* and *mev-1(kn1)*. We tested both mutants to determine if inhibited oxidative phosphorylation leads to hypoxia resistance. We found that mutation of both *clk-1* and *mev-1* results in strong resistance to hypoxia (Fig 7A). Therefore, reduced oxidative phosphorylation could also contribute to the hypoxia super resistance observed in day 3 adults. In support of this hypothesis, we found that repression of oxidative phosphorylation genes was partially suppressed in a *daf-16(mu86);glp-1(e2141)* double mutant (S3 Fig).

### Mutation of protein translation factors suppresses hypoxia “super resistance”

The results of our RNAseq analyses imply that reduced expression of translation genes may be responsible for the primary hypoxia resistance observed in both *glp-1(e2141)* and *fem-3(q20)* mutants and that repression of cytosolic ribosomal protein genes may be necessary for the super-resistance observed specifically in the *glp-1(e2141)* mutant as it ages past day 1 of adulthood.

In a previous study it was found that mutation of two genes, *ncl-1* and *lrp-1*, was able to suppress the hypoxia resistance of multiple protein translation mutants. Specifically, mutation of both of these genes together led to an increase in the abundance of cytosolic ribosomal proteins as determined by proteomic analysis [7]. To determine if *ncl-1* and *larp-1* mutations can suppress the hypoxia resistance of germline mutants, we generated the following triple mutant strains: *larp-1(q783);ncl-1(gc53);glp-1(e2141)* and *larp-1(q783);ncl-1(gc53);fem-3(q20)*. These strains were tested for hypoxia resistance as L4 larvae, day 1 adults, and day 2 adults and compared to *glp-1(e2141)* and *fem-3(q20)* single mutants. Interestingly, we found that dual mutation of *ncl-1* and *larp-1* did not suppress the day 1 primary hypoxia resistance of *fem-3(q20)* or *glp-1(e2141)* adults (Fig 7B and 7C). However, mutation of *ncl-1* and *larp-1* together did completely suppress the aging dependent increase in hypoxia resistance observed in *glp-1(e2141)* mutants, confirming that this aging dependent hypoxia super-resistance is likely due to reduced expression of genes involved in protein translation, particularly cytosolic ribosomal protein genes.

### *Glp-1* mutants are resistant to heat stress as adults but not l4 larvae

Our results demonstrate that the hypoxia resistant state of *glp-1(e2141)* doesn’t manifest until adulthood and becomes stronger as *glp-1(e2141)* mutants age. Thus, *glp-1(e2141)* L4 larvae undergo physiological changes between L4 and adulthood that confer strong resistance to hypoxic injury. This raises the possibility that the physiological properties that make *glp-1(e2141)* mutants long lived may also not occur until adulthood. This question is not easily addressed however, because it is impossible to comparatively age an L4 larva and adult. Resistance to heat stress is a marker that often correlates with longevity. It has previously been shown that the *glp-1(e2141)* mutant is increasingly resistant to heat stress as adults [19]. Therefore, we tested larval and adult worms to determine if the *glp-1(e2141)* and *fem-3(q20)* mutants were also resistant to heat stress in a developmentally dependent manner. Worms grown at room temperature to the appropriate stage were placed at 35°C for 10.5 hours and rescued by returning to 20°C. Surviving worms were recorded 24 hours later. We found that wild type worms, the *daf-16* single mutant, and a *larp-1;ncl-1* double mutant displayed little resistance to heat stress as L4 larvae, day 1 adults or day 2 adults (Fig 7D). In contrast, *glp-1(e2141)* mutants were only mildly resistant to heat stress as L4 larvae, but became more resistant to heat stress when reaching day 1 of adulthood. The heat resistance of *glp-1(e2141)* became even stronger when reaching day 2 of adulthood. Like hypoxia resistance, the aging dependent increase in resistance to heat stress was almost fully suppressed by mutation of *daf-16* and partially suppressed by mutation of *ncl-1* and *larp-1* together (Fig 7D). In contrast, *fem-3(q20)* adults, while resistant to hypoxic injury, displayed little to no resistance to heat stress. Taken together, our results demonstrate that the primary hypoxia resistance mechanism observed in both *fem-3(q20)* and *glp-1(e2141)* mutants does not correlate with resistance to heat stress, however the *daf-16* dependent hypoxia super-resistance observed in germline deficient animals does correlate well with resistance to heat stress and, therefore, may also be related to longevity.

## Discussion

### Hypoxia resistance correlates with reproductive output

Sterile mutations are sufficient to promote hypoxia resistance in *C*. *elegans*, as long as they prevent oogenesis or an upstream step in germline development [10]. For this study, we set out to further establish the mechanisms by which inhibition of reproduction brings about hypoxia resistance. Using FUDR to induce partial reductions in fertility we demonstrated that total sterility is not required for hypoxia resistance. In fact, hypoxia resistance is proportional to reproductive output. The connection between fertility and hypoxia resistance is an important one because previous mutagenic and RNAi screens in *C*. *elegans* have identified over 200 genes that, when suppressed, lead to hypoxia resistance [1]. It was reported that inhibition of many of these genes also leads to a partial reduction in progeny production [1]. Because reproduction is an energy intensive process, the majority of a worm’s metabolic output is devoted to the production of offspring. It therefore follows that many genes that facilitate hypoxia resistance may do so simply because they restrict the amount of energy and resources available for reproduction and thereby reduce the amount of oogenesis. Our results demonstrate that this possibility should always be considered when investigating how genes influence hypoxic injury.

### A hypoxia sensitive to hypoxia resistant transformation in sterile mutants

Since mutations that prevent oogenesis cause robust resistance to hypoxic injury, it is reasonable to postulate that it is the mechanism of oogenesis itself that renders worms more sensitive to hypoxia exposure. This hypothesis is not true, however, as we found that early L4 larvae that have not yet started oogenesis and WT males are just as sensitive to hypoxia as WT hermaphrodites. Furthermore, we found that *glp-1(e2141)* and *fem-3(q20)* L4 larvae, which also do not possess oocytes, are not resistant to hypoxic injury. Taken together, these data imply that it is the inhibition of oogenesis in otherwise oogenic animals that leads to hypoxia resistance, not the absence of oogenesis *per se*.

Perhaps the most interesting result of our study is the fact that the robust hypoxia resistance of sterile mutants is only observed in adult animals. In just a 12-hour period, both *glp-1(e2141)* and *fem-3(q20)* mutants transform from fully hypoxia sensitive L4 larvae to highly resistant day 1 adults. These findings imply there are considerable physiological differences between L4 larvae and day 1 adults, even in the *glp-1(e2141)* mutant, which does not have an active germline. This result could be viewed as surprising, especially for the *glp-1(e2141)* mutant, because the most reasonable model for why WT worms are sensitive to hypoxia and the *glp-1(e2141)* mutant is not, is the presence of a germline in WT animals. In this hypothesis, it is the metabolically active germline that sends signals that make animals sensitive to hypoxia. This scenario is not true, however, as our results demonstrate that the germline itself is not required for making animals sensitive to hypoxia. Instead, we must conclude that there are developmental programs operating in somatic tissues that render animals sensitive or resistant to hypoxia in germline mutants. These metabolic programs may very well exist to support germline development if it were present or, alternatively, the hypoxia resistant metabolic state of sterile adults may occur as a response to the absence of the germline and/or oogenesis. In either case, these metabolic programs operate when the germline is not present. These signals may also operate in WT worms when the germline is present, however, it is possible in WT animals that distinct mechanisms of hypoxia resistance occur. This could be presumably true for partial sterility as well, in that partial sterility is accompanied by changes in the somatic tissues that render animals partially resistant to hypoxic injury.

### A second mechanism of hypoxia resistance operates in germline ablated animals

We also found that *glp-1(e2141)* animals, but not *fem-3(q20)* animals, became even more resistant to hypoxia as they aged to day 2 of adulthood. In fact, over a third of *glp-1(e2141)* day 2 adults survive as much as 44 hours of hypoxia at 26.5°C. This is remarkably strong hypoxia resistance compared to WT animals, of which less than 10% survive only 20 hours of hypoxia. For clarity, we have termed this aging dependent increase in hypoxia resistance “super-resistance”. There are two lines of evidence that the super-resistance of the aging *glp-1(e2141)* mutant is a second mechanism of hypoxia resistance that is additive with the primary hypoxia resistance mechanism that operates in both *fem-3(q20)* and *glp-1(e2141)* mutants. First, the primary hypoxia resistance mechanism occurs in both *glp-1(e2141)* and *fem-3(q20)* mutants, whereas the aging dependent super-resistance only operates in the *glp-1(e2141)* mutant. Second, the primary hypoxia resistance mechanism of both sterile mutants does not require *daf-16*, whereas the super-resistance of the *glp-1(e2141)* mutant is completely dependent on *daf-16*. Like the primary hypoxia resistance mechanism, the super resistant metabolic state must reflect a transformation in somatic tissues that occurs in the hours after *glp-1(e2141)* worms reach adulthood. Therefore, again, it is not the germline itself that determines hypoxia sensitivity, but instead it is metabolic programs that operate in the somatic tissues in response to an active or inactive germline.

### Both hypoxia resistance mechanisms prevent mitochondrial protein aggregation

Protein folding errors and protein aggregation may be a key factor in hypoxic injury. It has been established that hypoxia causes protein aggregation in the mitochondria [17,18]. Furthermore, inducing the mitochondrial unfolding response is protective against hypoxic injury. Although mitochondrial protein aggregation correlates well with hypoxia sensitivity, it is not yet known if this phenomenon is responsible for cellular toxicity. Many factors have been identified that protect against hypoxic injury, but it is not yet known if these factors prevent aggregation in the mitochondria. In this study, we found that both the *fem-3(q20)* and *glp-1(e2141)* mutations prevented protein aggregation in the mitochondria. Furthermore, day 2 *glp-1(e2141)* worms were more effective at preventing aggregation than day 1 *glp-1(e2141)* worms. Therefore, it appears that both mechanisms of hypoxia protection in sterile mutants are effective at preventing mitochondrial protein aggregation.

### Aging dependent co-regulation of protein translation genes in *glp-1(e2141)* mutants

Ribosomes are very large, complex and abundant molecules that consist of multiple RNAs and dozens of distinct polypeptides. For this reason, it is generally assumed that the genes that encode ribosomal proteins must be regulated together so that precise stoichiometries are achieved and ribosomes are then able to fold and assemble properly [20,21]. Incorrect stoichiometry and ribosomal mis-assembly could be consequential especially given how abundant ribosomes are in the cytosol and mitochondria. Our results here confirm that the ribosomal protein genes (RPGs) are indeed regulated tightly together, being repressed in germline ablated animals as they transition from L4 to day 1 adults and further repressed as they age to day 3 of adulthood. We also found that the mitochondrial RPGs (mitoRPGs) are repressed together under the same scenarios, arguing that there is coordination between cytosolic and mitochondrial protein translation. However, the difference in repression intensity between the cRPGs and mRPGs suggest that the mRPGs are repressed via a different, more powerful, mechanism.

Our study has demonstrated that it is not just the ribosomal protein genes that are co-regulated, numerous other factors involved in cytosolic and mitochondrial protein synthesis are also repressed to a comparable degree as cRPGs and mRPGs in the *glp-1(e2141)* mutant. These factors include all 33 of the cytosolic and mitochondrial tRNA synthetases, various other ribosomal factors, nucleolar genes, as well as genes encoding mitochondrial translation initiation and elongation factors. Interestingly, these other translation factors were repressed between L4 and day 1 of adulthood, but were generally not further repressed between day 1 and day 3 of adulthood. Therefore, it appears that an entire protein translation apparatus, including both the cytosolic and mitochondrial systems, is repressed in *glp-1(e2141)* animals as they transition from L4 larvae to day 1 adults and then the cytosolic and mitochondrial ribosomal protein genes are further repressed as day 1 adults age to day 3 of adulthood.

In addition to the co-regulation of genes involved in protein translation, we also found that all 13 mitochondrial protein translocases were repressed in germline ablated animals to precisely the same degree as mitochondrial RPGs. Such strict co-regulation argues that it is essential that the stoichiometry of the mitochondrial protein translocation complex be coordinated with stoichiometry of the mitochondrial protein translation machinery. Therefore, future studies examining the co-regulation of genes involved in translation should focus on finding regulators of all protein translation genes as well as genes involved in mitochondrial translocation. Importantly, the regulation of protein translation and translocation genes occurs independently of germline signals in germline mutants, as their repression occurs in animals with no germline.

### *Daf-16* mediated repression of crpgs is the mechanism of super-resistance

It is established that mutations that reduce protein translation lead to hypoxia resistance [7]. For this reason, the reduced expression of genes involved in protein translation we observed in our RNAseq studies was a good candidate for the mechanism of hypoxia resistance in germline mutants. Furthermore, it is established that protein translation genes are broadly repressed in *glp-1(e2141)* adults vs WT worms, and this repression is dependent upon *daf-16* [22]. Here we provided two key pieces of evidence that the hypoxia super resistance observed in post day 1 adults is attributable to reduced cytosolic protein translation, more specifically to the repression of cytosolic ribosomal protein genes. First, we found that mutation of *daf-16* completely suppresses the hypoxia super-resistance mechanism. Using RNAseq, we demonstrated that mutation of *daf-16* specifically blocked the aging dependent repression of cytosolic ribosomal protein genes, and partially blocked the repression of cytosolic tRNA synthetases, but did not impact the repression of other protein translation genes, including the mRPGs, mitochondrial tRNA synthetases, and mitochondrial translocases[23]. Therefore, mutation of *daf-16* specifically impacts the transcriptional regulation of cytosolic translation genes. Second, we found that mutation of *ncl-1* and *larp-1* together, two repressors of protein translation, was able to suppress the hypoxia super-resistance of the *glp-1(e2141*) mutant. Interestingly, previous studies have demonstrated that dual mutation of *ncl-1* and *larp-1* also leads to an increase in cytosolic ribosomal proteins. Together, these findings demonstrate that the regulation of cRPGs is a key factor in hypoxia resistance, targeted by key regulators such as *daf-16*, *ncl-1* and *larp-1*.

Reduced protein translation has also been linked to the longevity of the *glp-1(e2141)* mutant [13]. It was found that the nucleoli of different long-lived strains, including *glp-1(e2141)*, are smaller than in WT worms [12]. Protein analysis determined that there were fewer ribosomal proteins present in *glp-1(e2141)* adults than there were in WT worms. Furthermore, mutation of *ncl-1* was able to suppress the longevity of *glp-1(e2141)* mutants [12]. Taken together with our results, it appears that the mechanism of hypoxia super-resistance is related, in some fashion, to the mechanism of longevity observed in *glp-1(e2141)* animals. This is further established by the fact that *daf-16* is able to suppress both longevity and super-resistance of *glp-1(e2141)* animals.

An interesting implication of our study is that the properties that enable *glp-1(e2141)* animals to live long may not be instituted in *glp-1(e2141)* worms until they reach adulthood. This hypothesis cannot be tested directly, however, because it is impossible to observe the aging of an L4 larvae without it first transforming into an adult. As a proxy to longevity, we did test the ability of larval and adult *glp-1(e2141)* animals to resist heat stress. We found that the *glp-1(e2141)* mutant is not resistant to heat stress as L4 larvae, but becomes resistant to heat stress as it reaches adulthood and becomes more resistant as it ages to day 2 of adulthood. These results correlate with hypoxia super-resistance. Therefore, if heat stress does indeed correlate with longevity, this would imply that only adult *glp-1(e2141)* animals contain the physiological properties that enable them to live longer lifespans. If this is true, it would establish the L4/adult transition as an excellent model for identifying the properties that enable worms to live extended lifespans.

### The mechanism of primary hypoxia resistance may also be reduced protein translation

Our study demonstrates that there is a profound transformation in both *glp-1(e2141)* and *fem-3(q20)* mutants between the L4 larval stage and adulthood, such that L4 larvae are fully sensitive to hypoxia injury whereas day 1 adults are highly resistant. This transition occurs in both *glp-1(e2141)* and *fem-3(q20)* adults. Using RNAseq, we found that cRPGs and mRPGs were also repressed in the *fem-3(q20*) mutant between L4 and day 1 of adulthood, only to a lesser degree than observed in the *glp-1(e2141)* mutant. This repression therefore correlates with observed hypoxia resistance, which is also weaker in the *fem-3(q20)* mutant. Furthermore, the fact that ribosomal protein genes were not further repressed in day 3 *fem-3(q20)* mutants also correlates with the observation that hypoxia resistance is not increased in *fem-3(q20)* animals after day 1 of adulthood. Thus, reduced protein translation could also be the mechanism of primary hypoxia resistance. However, the fact that mutation of *larp-1* and *ncl-1* does not impact primary hypoxia resistance in either *fem-3(q20)* or *glp-1(e2141)* mutants, and the fact that mutation of *daf-16*, which suppresses the repression of cRPGs in both day 1 and day 3 adults, only partially suppresses the primary hypoxia resistance argues that cytosolic RPGs are probably not involved in primary hypoxia resistance. It remains possible, however, that the repression of mitochondrial RPGs and other translation factors provides an explanation for the primary hypoxia resistance. More study is required to address this possibility.

### Reduced oxidative phosphorylation may also contribute to hypoxia resistance

We also found that genes involved in oxidative phosphorylation were repressed in *glp-1(e2141*) animals between day 1 and day 3 of adulthood, but were not repressed in *fem-3(q20)* adults. This raised the possibility that reduced oxidative phosphorylation may contribute to hypoxia resistance, specifically to the super resistance that occurs between day 1 and day 3 of adulthood. Consistent with this possibility we demonstrated that mutation of *clk-1* or *mev-1*, two genes involved in the oxidative phosphorylation mechanism, resulted in strong hypoxia resistance. The fact that mutation of *daf-16* partially suppressed the repression of oxidative phosphorylation genes supports the hypothesis that oxidative phosphorylation also contributes to hypoxia super resistance.

### The l4/adult transition as a system for studying the unique properties of germline mutants

In this study, we have determined that the hypoxia resistance of germline deficient mutants is not a general property, but instead hypoxia resistance is “switched on” only in adult animals. This observation demonstrates that the signals that determine hypoxia sensitivity in germline mutants are derived from somatic tissues and act in developmental fashion. This study therefore pinpoints the L4/adult transition of germline deficient mutants as an excellent system to study the unique physiological properties of germline mutants. Adult germline mutants can simply be compared to L4 larvae to identify physiological changes that determine hypoxia sensitivity. This is a much cleaner system than comparing germline deficient mutants to WT animals. In this study, we used such a comparison to identify reduced expression of genes involved in protein translation as one of the mechanisms of hypoxia resistance in germline deficient animals. Our data also suggest that this same phenomenon may be true for the long-lived properties of germline deficient animals. Future studies should focus on the L4/adult transition to identify physiological changes associated with hypoxia resistance, heat stress resistance, and longevity.

## Methods

### *C*. *elegans* strains and maintenance

**N2-Bristol**, **AGD1032:***glp-1(e2141)*, **CF1880:***daf-16(mu86);glp-1(e2141)*, **CB3844:***fem-3(e2006)*, **JK816:***fem-3(q20)*, **SS104:***glp-4(bn2)*, **TK22**:*mevl-1(kn1)* and **MQ130**:*clk-1(e2519)* were obtained from the *C*. *elegans* Genetics Center at the University of Minnesota. **MC893:***larp-1(q783);ncl-1(gc53)* was obtained as described. The following compound mutant strains were constructed using standard genetic techniques: **MC952:***larp-1(q783);ncl-1(gc53);glp-1(e2141)*, **MC942:***larp-1(q783);ncl-1(gc53);fem-3(q20)*, and **MC985:***daf-16(mu86);fem-3(q20)*.

Animals were maintained at 20°C or 25°C on nematode growth media (NGM) plates seeded with Escherichia coli OP50. The N2 (Bristol) strain was the standard wild type strain from the *C*. *elegans* Genetics Center (CGC, University of Minnesota). Compound mutants were constructed using standard genetic techniques. Genotypes were confirmed by PCR amplification or by PCR followed by restriction digest.

### Hypoxia assays

For hypoxia assays, worms were synchronized by allowing 5 hermaphrodites to lay eggs for 4 hours. Adult hermaphrodites were removed from plates and synchronized progeny were grown at 25°C until ready for assay. Worms were assayed as either L4 larvae, day 1 adults or day 2 adults. Day 1 adults were assayed ~12–24 hours after the L4/adult transition, day 2 adults were assayed 36–48 hours after the L4/adult transition. Synchronized young adult worms were subjected to hypoxia as described previously except that hypoxic incubation temperature was 26.5°C [24]. Briefly, each plate of worms was washed into one 1.5 ml tube with 1 ml of M9 buffer (22 mM KH2PO4, 22 mM Na2HPO4, 85 mM NaCl, 1 mM MgSO4). Worms were allowed to settle by gravity, and 900 μl of M9 buffer was removed. The tubes were then placed in the anaerobic chamber (Forma Scientific) at 26.5°C for the indicated incubation times. Oxygen tension was always ≤ 0.3%. Following the hypoxic insult, worms were placed using glass Pasteur pipettes onto NGM plates spotted with OP50 bacteria and recovered at 20°C for 24 hours. Worms that moved or responded to being prodded with a pick were scored as alive.

### FUDR assays

Worms were synchronized by allowing 5 hermaphrodites to lay eggs for 4 hours. When progeny reached the L4 stage of development, the appropriate concentration of FUDR was added to the growth plates. Worms were then grown to the appropriate stage, placed in hypoxia chamber and assayed as described above.

### Analysis of protein aggregation

Wild type and mutant worms containing the *ucr-11*::*gfp* reporter construct were synchronized as described above and grown to day 1 or day 2 of adulthood. Worms were then exposed to hypoxia at 26.5°C. Immediately after hypoxia exposure, worms were mounted for microscopy. Confocal microscopy was performed using a Leica TCS SP8 equipped with a HyD detector. Paralysis was produced by mounting worms in a solution of 50 mM levamisole (Sigma-Aldrich Corp., St. Louis, MO, USA) in M9 prior to imaging. Images were acquired from at least ten randomly selected worms at 1024 × 1024 resolution using a 63 × objective with 8 × zoom producing a 23.07 × 23.07 *μ*m image (defined as one high power field, HPF). All images were acquired as a ten slice Z-stack with scan speed of 800–1800 Hz and flattened as a maximum intensity projection prior to analysis. Analysis of images was performed by an observer blinded to condition. For assessment of total fluorescence images were acquired on a Zeiss Axioskop2 microscope with fluorescence intensity quantified using NIH ImageJ.

### Heat stress assays

Worms were synchronized by allowing 5 adults lay eggs for 4 hours. Synchronized progeny were grown to the L4, day 1, or day 2 stage of adulthood on NGM plates with OP50 and then were placed in a 35°C incubator for 10.5 hours. Worms were then returned to 20°C and scored for survival 24 hours later.

### RNAseq

*C*. *elegans* strains *glp-1(e2141)* and *fem-3(q20)* were grown at 20°C on high-growth plates seeded with OP50 bacteria and maintained as described [25]. Gravid adults from 10 10-cm plates were bleached, and embryos were dispersed onto 15-cm nematode growth media (NGM)-lite plates seeded with OP50 and then grown at 25°C until the appropriate stage. ~10,000 Worms at the L4 stage, day 1 stage and day 3 stage were harvested, washed twice with M9, and frozen in liquid nitrogen. For RNA preparation, worms were thawed at 65°C for 10 min, and RNA was isolated using the Tri-Reagent Kit (Molecular Research Center, Cincinnati, Ohio, United States). RNAseq was performed by NovoGene Corporation, Sacramento, California. RNAseq files from NovoGene were processed by Seattle Genomics, Seattle Washington. Data were processed as follows: we performed adapter removal and quality trimming on the raw sequencing reads using Trim Galore, a wrapper of cutadapt (Martin 2011). We then mapped reads to the Ensembl release 104 *C*. *elegans* genome (WBcel235) using STAR (Dobin et al. 2013). Gene counts were quantified from the aligned reads using htseq-count (Anders & Huber 2015). Count files were imported into the R statistical computing software for all analyses. We first removed genes with counts less than 10 averaged across all samples. The filtered count matrix then underwent TMM normalization (Robinson & Oshlack 2010) followed by voom transformation (Law et al. 2014). These methods created a file listing all *C*. *elegans* genes by log fold change (Log FC) (S1 Table).

For initial differential expression testing (S2 and S3 Fig), testing was performed using limma. A gene was considered significantly differentially expressed with an adjusted p-value < 0.01 and an absolute fold change > 2. Differentially expressed genes were binned into co-expression clusters using the R packages heatmap.2 and WGCNA (Langfelder & Horvath 2008). We tested each co-expression cluster for enrichment among Gene Ontology categories and KEGG pathways using WebGestaltR (Liao et al. 2019).

For analysis of translation genes, normalized log FC (fold-change) values from differential expression files were isolated for all *C*. *elegans* translation genes, including genes repressed by less than 2-fold (S1–S4 Tables). For histogram plots, the number of RPGs within each bin of repression were plotted. For example, there were X receptors in a bin representing the Log FC range of -1.0 to -0.9, and Y number of receptors in a bin representing Log FC -0.9 to -0.8, etc. Log FC values were converted to fold change for presentation. Statistical significance was determined using a T-test for two independent means.

## Supporting information

S1 Fig Hypoxia resistance in aging N2, *glp-1(e2141)* and *fem(q20)* mutants. Percent of animals surviving a 40-hour hypoxia exposure is shown.
(TIFF)

S2 Fig Regulation of protein translation genes in *glp-1(e2141), fem-3(q20)* and *daf-16(mu86);glp-1(e2141)* mutants. Genes displayed represent a partial list of genes involved in protein translation. Red rectangles represent higher levels of expression and blue colored rectangles represent lower levels of gene expression. Data are shown for L4 larvae, day 1 adults and day 3 adults for all three mutants. Each vertical row represents an individual RNAseq biological replicate. Data shows that nearly all displayed translation genes are repressed in the *glp-1(e2141)* mutant between L4 and day 1 of adulthood. Some translation genes, particularly cytosolic and mitochondrial ribosomal protein genes are repressed even more robustly in *glp-1(e2141)* day 3 adults. In the *fem-3(q20)* mutant, most translation genes are not repressed in day 1 or day 3 adults, however cytosolic and mitochondrial ribosomal protein genes are partially repressed in day 1 adults, but not further repressed in day 3 adults. While most translation genes are not impacted by mutation of *daf-16*, mutation of *daf-16* does specifically suppress the repression of cytosolic ribosomal protein genes in both day 1 and day 3 adults.
(TIFF)

S3 Fig Regulation of oxidative phosphorylation genes in *glp-1(e2141), fem-3(q20)* and *daf-16(mu86);glp-1(e2141)* mutants. Red represents higher levels of expression and blue represents repression. Expression of numerous oxidative phosphorylation genes is repressed between L4 and day 3 of adulthood in the *glp-1(e2141)* mutant, but not in the *fem-3(q20)* mutant. Mutation of *daf-16* partially suppresses the repression of some oxidative phosphorylation genes in the *glp-1(e2141)* background.
(TIFF)

S1 Table Differential Expression Analysis of *glp-1(e2141), fem-3(q20)*, and *daf-16(mu86);glp-1(e2141)* mutants between L4 and day 1 of adulthood, or L4 and day 3 of adulthood. (XLSX)

S2 Table Log FC values for cytosolic ribosomal protein genes in *glp-1(e2141), fem-3(q20)* and *daf-16(mu86);glp-1(e2141)* mutants. (XLSX)

S3 Table Log FC values for mitochondrial ribosomal protein genes in *glp-1(e2141), fem-3(q20)* and *daf-16(mu86);glp-1(e2141)* mutants. (XLSX)

S4 Table Log FC values tRNA synthetases, nucleolar genes, and mitochondrial translocases in glp-1(e2141), fem-3(q20) and daf-16(mu86);glp-1(e2141) mutants. (XLSX)
