# Supplementary figures and images for: Daf-16 mediated repression of cytosolic ribosomal protein genes facilitates a hypoxia sensitive to hypoxia resistant transformation in long-lived germline mutants
Source: PLoS Genet. 2022 May 27;18(5):e1009672. doi: 10.1371/journal.pgen.1009672 (PMC9197040; doi:10.1371/journal.pgen.1009672)

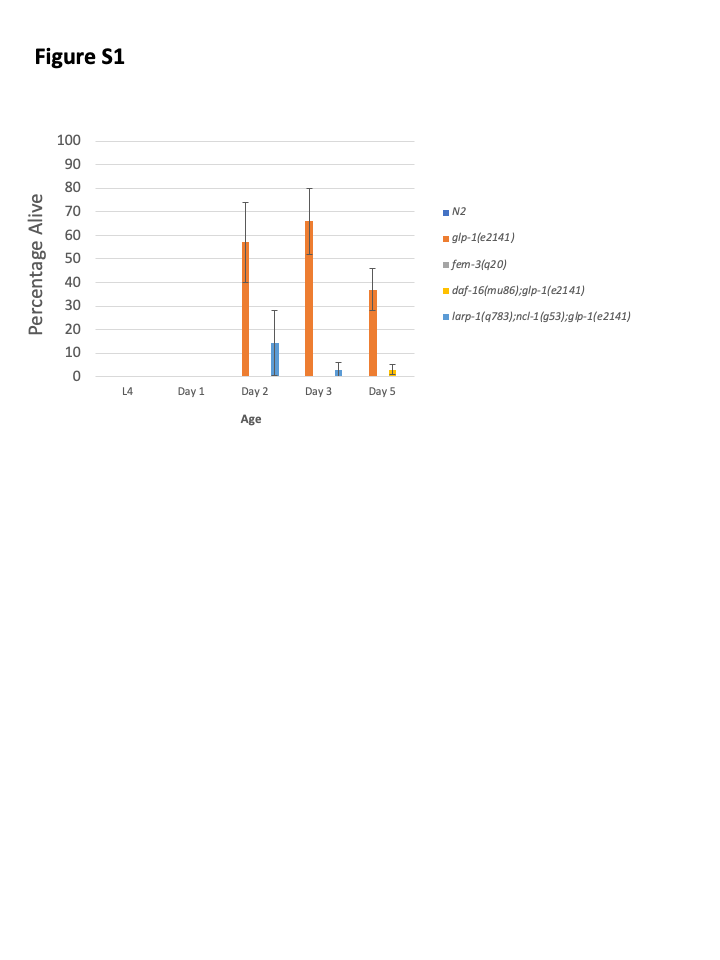

Supplement: S1 Fig — Percent of animals surviving a 40-hour hypoxia exposure is shown. (TIFF) [file pgen.1009672.s001.tiff]

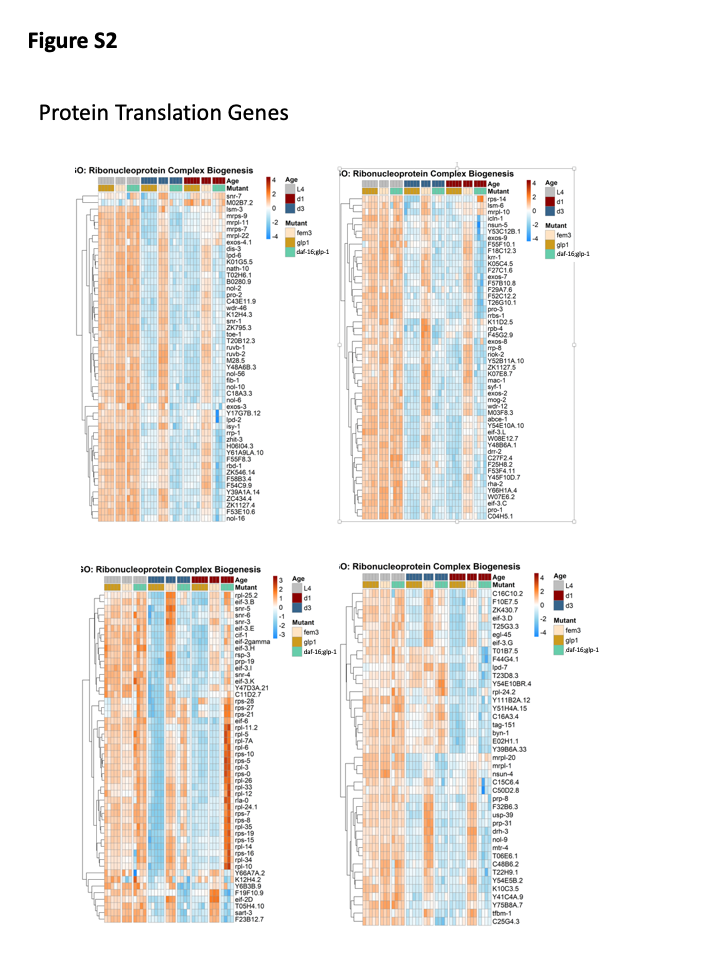

Supplement: S2 Fig — Genes displayed represent a partial list of genes involved in protein translation. Red rectangles represent higher levels of expression and blue colored rectangles represent lower levels of gene expression. Data are shown for L4 larvae, day 1 adults and day 3 adults for all three mutants. Each vertical row represents an individual RNAseq biological replicate. Data shows that nearly all displayed translation genes are repressed in the glp-1(e2141) mutant between L4 and day 1 of adulthood. Some translation genes, particularly cytosolic and mitochondrial ribosomal protein genes are repressed even more robustly in glp-1(e2141) day 3 adults. In the fem-3(q20) mutant, most translation genes are not repressed in day 1 or day 3 adults, however cytosolic and mitochondrial ribosomal protein genes are partially repressed in day 1 adults, but not further repressed in day 3 adults. While most translation genes are not impacted by mutation of daf-16, mutation of daf-16 does specifically suppress the repression of cytosolic ribosomal protein genes in both day 1 and day 3 adults. (TIFF) [file pgen.1009672.s002.tiff]

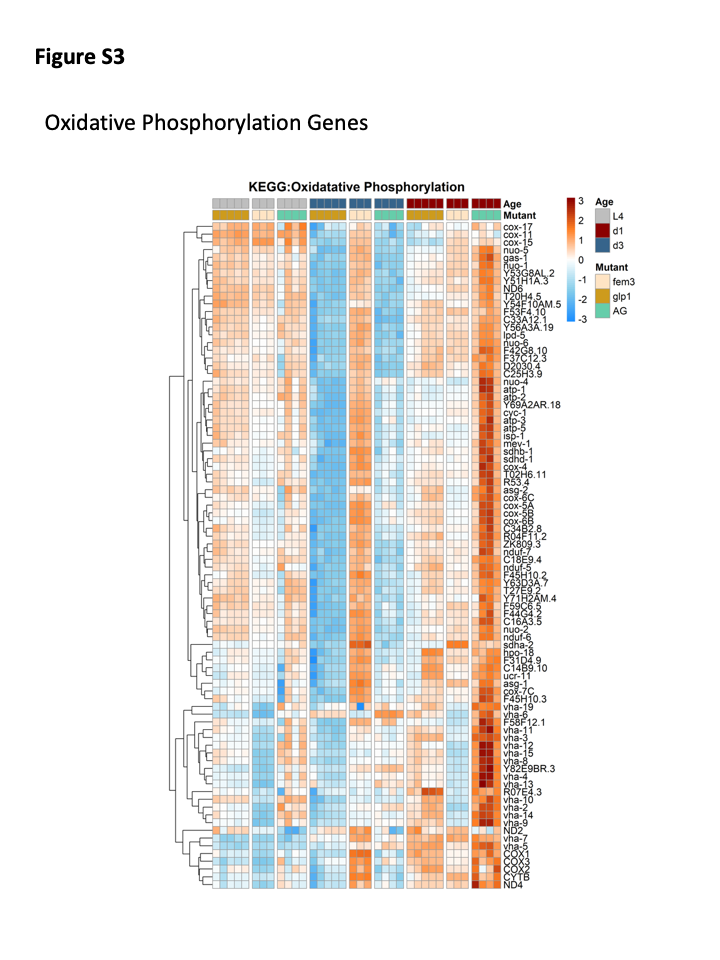

Supplement: S3 Fig — Red represents higher levels of expression and blue represents repression. Expression of numerous oxidative phosphorylation genes is repressed between L4 and day 3 of adulthood in the glp-1(e2141) mutant, but not in the fem-3(q20) mutant. Mutation of daf-16 partially suppresses the repression of some oxidative phosphorylation genes in the glp-1(e2141) background. (TIFF) [file pgen.1009672.s003.tiff]
